# Nanomaterials for biogas augmentation towards renewable and sustainable energy production: A critical review

**DOI:** 10.3389/fbioe.2022.868454

**Published:** 2022-09-02

**Authors:** Sohaib Z. Khan, Asad A. Zaidi, Muhammad Nihal Naseer, Hamad AlMohamadi

**Affiliations:** ^1^ Department of Mechanical Engineering, Faculty of Engineering, Islamic University of Madina, Madinah, Saudi Arabia; ^2^ Department of Mechanical Engineering, Faculty of Engineering Science and Technology, Hamdard University, Karachi, Pakistan; ^3^ Department of Engineering Sciences, PN Engineering College, National University of Sciences and Technology, Karachi, Pakistan; ^4^ Department of Chemical Engineering, Faculty of Engineering, Islamic University of Madinah, Madinah, Saudi Arabia

**Keywords:** anaerobic fermentation, biogas, nanotechnology, nanoparticles (NPS), waste, biomass, biohydrogen, nanomaterial

## Abstract

Nanotechnology is considered one of the most significant advancements in science and technology over the last few decades. However, the contemporary use of nanomaterials in bioenergy production is very deficient. This study evaluates the application of nanomaterials for biogas production from different kinds of waste. A state-of-the-art comprehensive review is carried out to elaborate on the deployment of different categories of nano-additives (metal oxides, zero-valent metals, various compounds, carbon-based nanomaterials, nano-composites, and nano-ash) in several kinds of biodegradable waste, including cattle manure, wastewater sludge, municipal solid waste, lake sediments, and sanitary landfills. This study discusses the pros and cons of nano-additives on biogas production from the anaerobic digestion process. Several all-inclusive tables are presented to appraise the literature on different nanomaterials used for biogas production from biomass. Future perspectives to increase biogas production via nano-additives are presented, and the conclusion is drawn on the productivity of biogas based on various nanomaterials. A qualitative review of relevant literature published in the last 50 years is conducted using the bibliometric technique for the first time in literature. About 14,000 research articles are included in this analysis, indexed on the Web of Science. The analysis revealed that the last decade (2010–20) was the golden era for biogas literature, as 84.4% of total publications were published in this timeline. Moreover, it was observed that nanomaterials had revolutionized the field of anaerobic digestion, methane production, and waste activated sludge; and are currently the central pivot of the research community. The toxicity of nanomaterials adversely affects anaerobic bacteria; therefore, using bioactive nanomaterials is emerging as the best alternative. Conducting optimization studies by varying substrate and nanomaterials’ size, concentration and shape is still a field. Furthermore, collecting and disposing nanomaterials at the end of the anaerobic process is a critical environmental challenge to technology implementation that needs to be addressed before the nanomaterials assisted anaerobic process could pave its path to the large-scale industrial sector.

## Introduction

Exponential growth in the world population has raised the energy demand drastically (Hagos et al., 2017). Meeting the energy requirement has now become an area of prime importance for all nations. At present, the world is highly dependent on conventional energy sources, i.e., fossil fuels (Palaniappan, 2017). The available reserves for fossil fuels are diminishing rapidly; one study indicated that existing reserves would last till 2050 (Satyanarayana et al., 2011). Besides, these conventional fuels contribute much to environmental pollution and ecological destruction. Along with fluctuating fuel prices, these factors have led the fuel industry to move towards sustainable renewable resources to fulfill the energy demand (Malik and Sangwan, 2012). Currently, fossil fuels fulfill almost 90% of world energy demands, and it is expected to minimize it to 50% by 2040 via incorporating more sustainable renewable energy sources such as solar, wind, geothermal, tidal, and biomass (biofuels) (Hussein, 2015).

Biofuels can be produced by utilizing locally available organic feedstock. Various methods are available for organic matter to energy conversion, but AD (Anaerobic Digestion) is among the most preferable, specifically for biogas production (Hao et al., 2019; Feng et al., 2021). In this process, the absence of O_2_ provides a favorable environment for bacteria to decompose organic matter by breaking it into methane and other by-products (Seadi et al., 2008). AD finds its implications for waste treatment on a broad category of waste, including sludge, wastewater, and municipal waste (Vasco-Correa et al., 2018). It is also mentioned among widely considered methods for converting complex waste to biogas (Holm-Nielsen et al., 2009; Feng et al., 2014). Additionally, applications of AD in the treatment of animal manure (Bidart et al., 2014), energy crops (Lönnqvist et al., 2013), organic food waste (Zhang et al., 2016), microalgae (Park et al., 2009), and agricultural residues (Mushtaq et al., 2016) make it stand among other methods.

**FIGURE 1:**
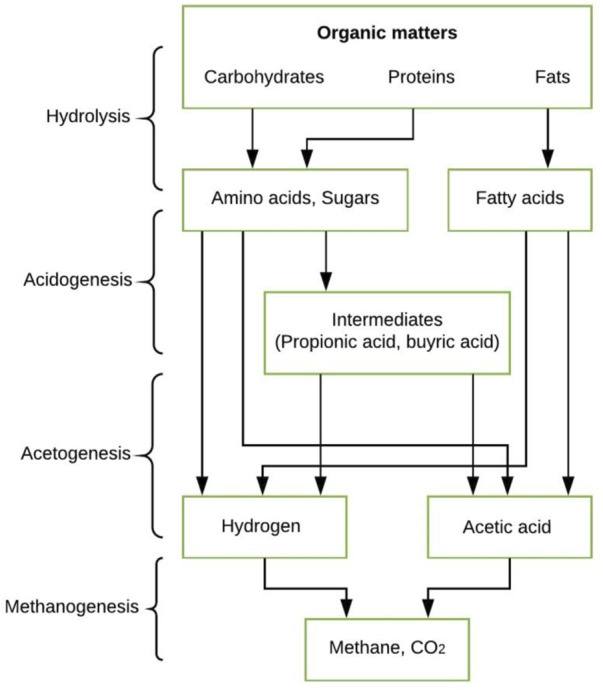
Common major sequential processes during anaerobic digestion (Feng et al., 2018).

In the mentioned process of organic waste conversion to biogas, four main phases are usually included; (i) hydrolysis, (ii) acidogenesis, (iii) acetogenesis, (iv) methanogenesis (Christy et al., 2014), see Figure 1. These four phases highly dependent upon the extent of interactions between microorganisms during each phase. In the first phase, hydrolytic bacteria are in action. They transform complex organic matters such as proteins, fats, and carbohydrates into organic monomers. Most organic matters contain complex macromolecules that cannot be directly used by acidogenic microorganisms. Therefore, hydrolysis is needed to break complex structures into small molecules (monomers), which ultimately can be used in the second phase of anaerobic digestion. In the second stage, acidogenesis, thus formed monomers are transformed into Volatile Fatty Acids (VFAs) with the help of fermentative bacteria. In the third phase, acetic acid is formed accompanied by evolving hydrogen gas by the action of acetogenic bacteria. Among four phases of anaerobic digestion, acidogenesis is considered the fastest one. The last stage is methanogenesis, where products of the last phase are transformed into methane and carbon dioxide (Mao et al., 2015; Zaidi et al., 2021a). Thus, formed methane significantly varies in quality based on a few factors such as biomass composition, additives, selection of conversion process, and precursors. Typically, the composition of biogas is specified by methane and carbon dioxide contributing 50–75% and 25–45%, respectively. A minute amount of other gasses can be there, usually of calorific values of 21–24 MJ/m^3^ (Ganzoury and Allam, 2015).

**FIGURE 2:**
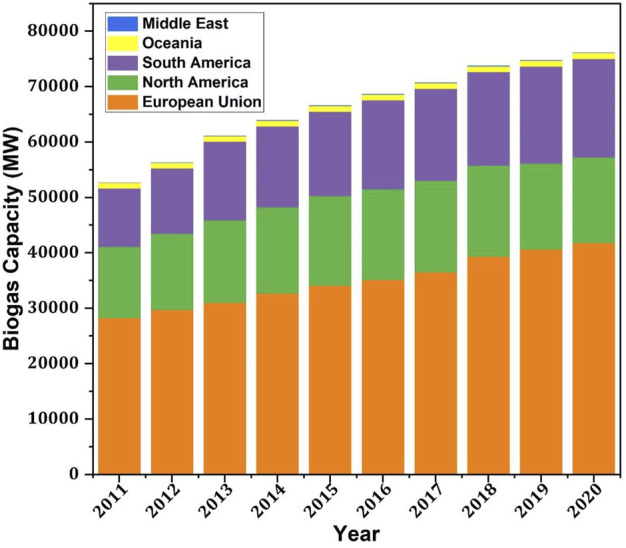
Regional breakdown of global biogas capacity (RENA, 2021).

Biogas, as a renewable energy source, is an emerging sector globally with consecutive increments in the production capacity over the years. Figure 2 represents the regional breakdown, not only reflecting the overall increment but also every region is showing growth over the years, which is a promising motivation for scientists and investors for the biogas augmentation utilizing all the available technologies to pursue state-of-the-art solutions for biogas production. Nanotechnology, which can be defined as interpolation of matter at very small dimensions (less than or equal to 100 nm), is in its emerging phase. At this small scale, material properties change (such as melting point and chemical changes) that has made this technology pivot to researchers (Antonio et al., 2017). Nanotechnology can be used in many fields such as materials engineering, life sciences, electronics, biotechnology, information technology, and cognitive sciences (Khan et al., 2009; Demetzos, 2016). The bioenergy field can be revolutionized by improving catalytic conversions and enhancing catalytic efficiency. Literature is evident from the recent implications of nanoparticles (NPs), nanomaterials (NMs), nanosheets, and others in bioenergy production (Rahman et al., 2016). Wu et al. (2021) recently conducted a literature review to highlight the importance of different operating parameters on biogas production and to understand the importance of different auxiliary technologies in optimizing these operational parameters. The study finds that the addition of NPs is a promising option, especially for mainstream biogas production plants, to enhance biogas production. However, some challenges (such as high investment cost, strict control of NPs concentration, energy demand, and disposal risks) need to be minimized before introducing NPs in the industrial sector (Zaidi et al., 2021b). In another review study (Jadhav et al., 2021), the authors studied the impact of metallic NPs on microbial direct interspecies electron transfer for biogas production enhancement. The use of metallic NPs was found to be cost-effective, efficient, and sustainable for biogas production. Hassanein studied the role of electro-conductive NPs. NPs were found to be promising for AD process stability and efficiency enhancement (Zaidi et al., 2019a; Kumar et al., 2021). Specifically, metallic NPs were highlighted as the most famous NPs for their potential to decrease lag time and improve the biogas production and process stability. Moreover, studying the role of size, type, and concentration of metallic NPs is still a challenge (Hassanein et al., 2021). After conducting a literature review, Ellacuriage stipulated that to increase volumetric efficiency and reduce initial capital cost, NPs augmentation is the most suitable approach (Ellacuriaga et al., 2021).

The economic feasibility of large-scale AD has always been a prime concern for the research community. The application of NPs has contributed to the economic feasibility of AD by enhancing catalytic efficiency (Faisal et al., 2019). However, the disposal of these NPs after biogas production is still a significant environmental challenge. Therefore, there is a dire need to find environmentally friendly disposing methods for NPs being used in AD. Moreover, the main challenge in understanding nanomaterial’s augmentation with biogas is their kinetics. The root cause of lower biogas production in the absence of NPs is a cellular wall that restrains the interaction of catalysts with the substrate. Studying the impact of different NPs, through the lens of their positive and negative aspects could improve our understanding of biogas production.

This paper presents a comprehensive state-of-the-art review highlighting the direct influence of nano-additives and nano-nutrients on either biogas production enhancement or adverse effects during anaerobic digestion. Future perspectives to enhance biogas production via nano-additives are also presented. The focus has been placed on classifying available literature according to the type of nanomaterial employed during AD. The detailed discussion shows how nanomaterials can be effectively used for biogas augmentation to improve biomass utilization as a renewable and sustainable energy source. Furthermore, this study reports a bibliometric analysis of biogas literature published in the last 50 years. To the best of the authors’ knowledge, it is the first study based on a detailed quantitative literature review.

## Nanomaterials role in chemical reactions

Nanomaterials (NMs) are materials having one or more dimensions smaller than 100 nm. This resulted in a much high surface area of the material just because of the size. A spherical NP of 1 nm diameter will have approximately 100% of its atoms on the surface. Whereas an NP having a diameter of 10 nm would have only 15% of its atoms on the surface. It would be expected from a particle having a higher surface area to be more reactive than the same mass of material consisting of larger particles, as chemical reactions typically take place at surfaces (Rao et al., 2001).

NMs can be classified into three categories contingent on a number of dimensions at the nanoscale as per the British Standards Institution (BSI, 2007). Table 1 depicts some NMs from each group. In the literature, nanoparticles are specified as 3D particles having at least one dimension of less than 100 nm. They could have various morphologies and shapes. As discussed earlier, the surface properties and high reactivity of the NPs are due to the increased surface area to volume ratio. This distinctive feature of NPs makes them popular in products and techniques where chemical reactions are important. In this text, nanomaterials and nanoparticles are used as interchangeable terms, both referring to the nano-scale materials in the context of the discussion.

**TABLE 1:** Classification of nanomaterials.

Classification	Examples
One dimensional NMs	Nanolayers
Two dimensional or 2D NMs	Nanowire, nanotube, nanorod, Graphene
Three dimensional NMs	Quantum dots, fullerenes, metal and metal oxides NPs

There are numerous benefits of NMs for biogas production. NMs provide more exposed sites available for anaerobic bacteria (Rahman et al., 2016). It also helps in the solubilization of organic matter to release intercellular polymeric substances. The control over surface features aids in catalyzing animal fats, plant cell membranes, and cellular remains. They also help a chemical modification of organic matter (Nyberg et al., 2008). The application of NMs for biogas production can be one of the possible ways to sustain this renewable energy source for large-scale production. Several NMs are used as an additive to enhance biogas production.

## Research trends in biogas studies: Past and contemporary

**FIGURE 3:**
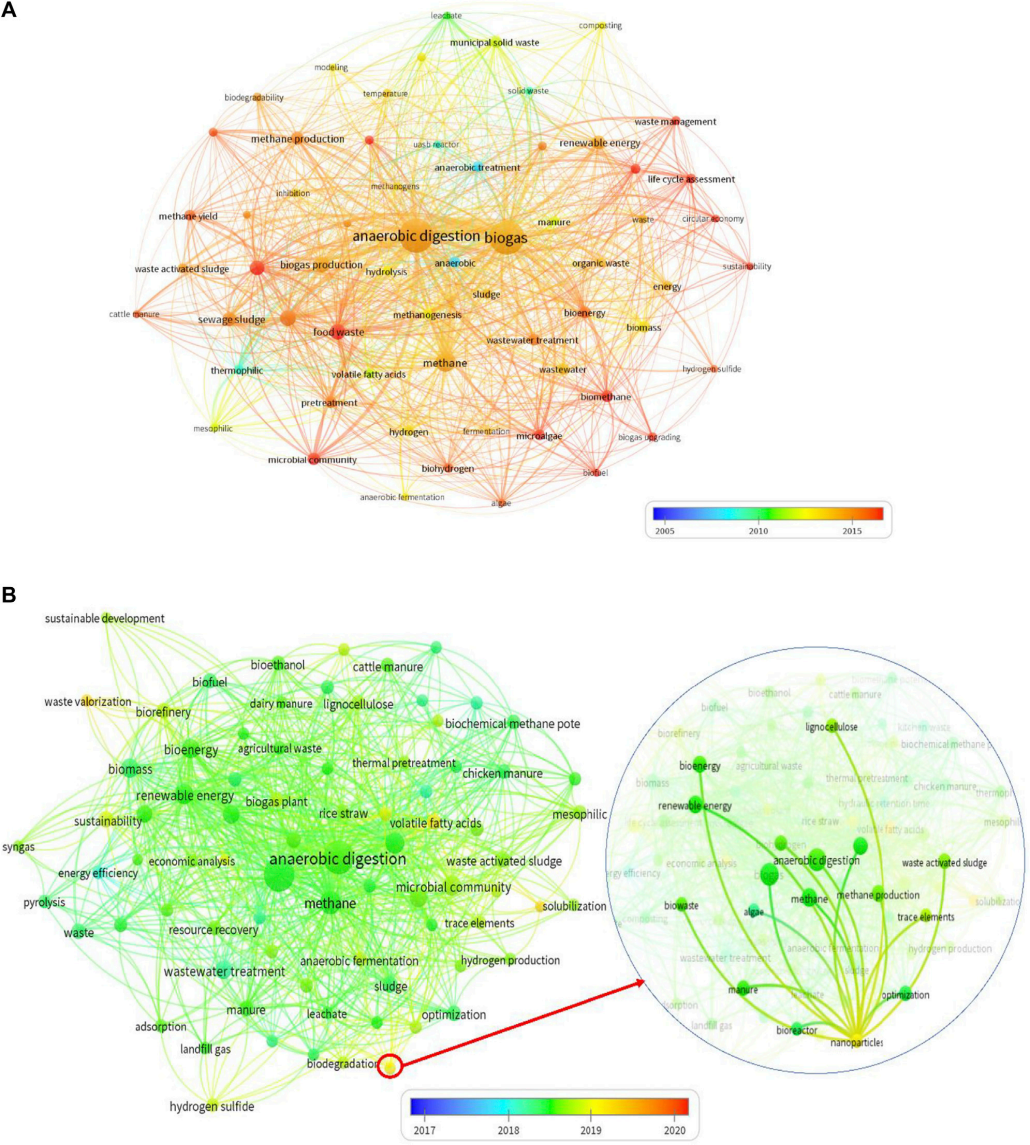
Major research terms used by researchers from **(A)** 1970–2020 **(B)** 2017–2020.

**FIGURE 4:**
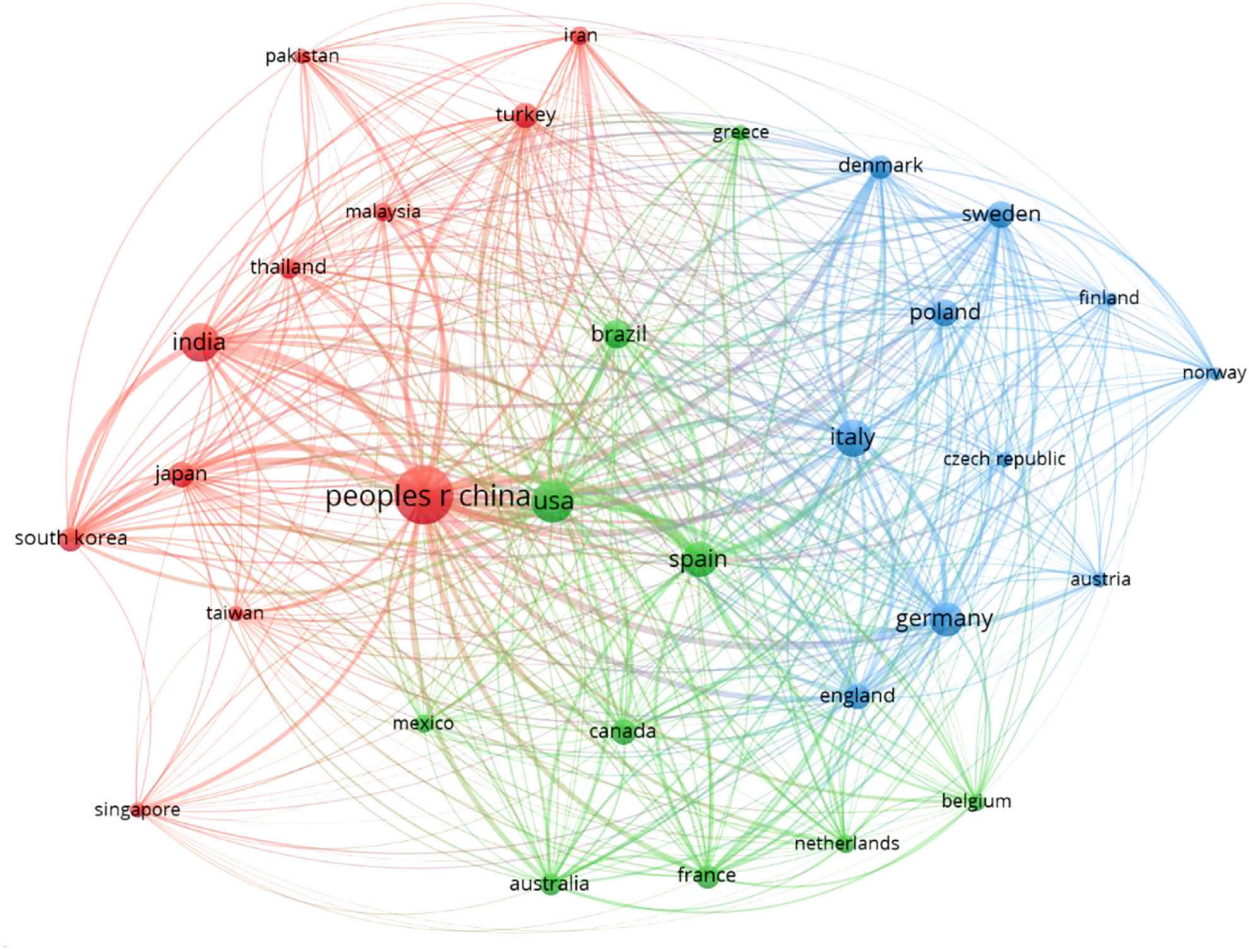
Currently active countries on nanotechnology-based biogas production on the basis of the number of citations.

In order to find out a pattern, sequence, and significant research trends, quantitative analysis is performed using the web of science database, as shown in Figures 3,4. To conduct the analysis, 14,000 journal articles (research papers only) were explored from the web of science database, and content analysis was performed to determine the main keywords used by researchers. These keywords define the mainstream of research within a field. The colors depict different eras of research. The diameter of bubbles denotes the impact of that keyword, i.e., the occurrence of a keyword. These bubbles are interconnected using links. Link strength is evident in the relation between two keywords, i.e., co-occurrence in the same research article.

The survey was divided into two eras for analysis purposes, the first 1970–2016 and the second 2017–2020. The purpose of this division was first to understand research evaluation within the field and second to determine the current topics of research to define future directions. Figure 3 revealed that anaerobic digestion and biogas production are among the most used keywords throughout the era 1970–2020. In addition, these keywords find their most implications in the last 5 years as denoted by red color. Therefore, it is concluded that anaerobic digestion and biogas production are among hot topics of research.

In order to further understand the main streams of research within anaerobic digestion and biogas production, data from the last 4 years were evaluated. It is pertinent to mention that 2010–2020 is observed as the main era of research rise in this field. A total of 84.4% of the publications have been published in the last 10 years. Out of this, 84.4%, 54.7% of publications belong to the last 4 years, 2017–2020. Therefore, 2017–2020 can be mentioned as a research-intensive period of biogas production. This high research interest is due to the emergence of new technologies and their implications for biogas production.

The analysis of research keywords used in the last 3 years depicts that the emergence of NP is the main technology that evolved in this era and got incredible attention from the research community. The yellow color of the keyword NP is evident to a sharp contrast and shift towards effective implementation of NP in producing biogas during 2019–2020. The strong link of NPs with anaerobic digestion, methane production, and waste activated sludge represents NPs’ reputation for mentioned technologies with in short duration. Owing to this reputation, NPs implications for biogas production can be regarded as the central pivot to the research community.

The most important aspect to note is the emergence of nanoparticles in the last decade and their strong connection with biogas production. Therefore, based on research trends, it can be concluded with confidence that nanoparticles and biogas production starting from sludge have gotten significant attention in recent years. In this regard, this review is conducted to update how nanomaterials have contributed to biogas production.

## Application of nanomaterials for biogas production

This section presents a comprehensive review of the recently reported studies on biogas production based on the class of materials used for a different kind of feedstock. Nanomaterials are a vital candidate to enhance biogas production from different inorganic waste. Basically, at the nanoscale, the surface area of the material is high, making the reaction relatively fast (Zaidi et al., 2019a). In addition, these NPs interact with the cell membrane of sludge, leading to structural changes in the cells that finally make it bacteria permeable membranes. In this way, more bacteria find their way to attack sludge and hence increase overall biogas production (Faisal et al., 2019). Nevertheless, attention has been focused on the use, effects, and outcomes of various NMs for biogas production.

### Trace metal nanomaterials for biogas enhancement

Trace metals are essential for methanogenic bacteria growth in an AD reactor (Qiang et al., 2013). Metals nutrients such as iron, cobalt, nickel, etc., are found to influence the AD process significantly (Kelly and Switzenbaum, 1984; Zaidi et al., 2018). Zero-valent iron has been widely employed to treat various kinds of waste. The literature showed that it releases electrons for methanogenesis during the AD process, resulting in biogas augmentation. Nanoscaled Zero-Valent Iron (NZVI) has a high surface-to-volume ratio; this characteristic increased the chemical reaction sites and positively influenced the AD. Su et al. (2015) investigated the influence of 0.05, 0.10, and 0.20 wt% NZVI (60–120 nm) on the AD of Waste Activated Sludge (WAS) for 20 days at the mesophilic temperature (32 ± 1°C). The results indicated that 0.05 wt% and 0.10 wt% NZVI increased the methane production by 9.8% and 4.6%, respectively. However, 0.20 wt% NZVI decreased methane production by 8.8%. The authors suggested that NZVI stimulates methanogenic populations and sulfate reducers. It also accelerates sludge stabilization in AD resulting in increased biogas and methane production. The metallic iron core caused a slow release of soluble Fe^2+^ acting as a donor and caused the formation of reactive oxygen species. The hydrogen sulfide reacted with NZVI oxide shell on the surface and resulted in the formation of FeS and FeS_2_, which was regarded as the main reason for decreasing H_2_S and an increase in methane. These findings agree with Carpenter et al. (2015), who reported that cytotoxicity of NZVI to the microorganism in the AD with varied particle size and reactivity could improve the degradation increase biogas production while decreasing CO_2_. The observed decrease in biogas production at a higher concentration of NZVI by Su et al. (2015) was confirmed by the study conducted by Suanon et al. (2016). According to the authors, improvement in biogas and methane production is dose-dependent, and a higher dose of NZVI could result in an inhibitory effect. Another study conducted by Suanon et al. (2017) investigated the effect of 0.1 wt% NZVI on methane yield from wastewater sludge at mesophilic conditions (37 ± 1°C) for 50 days. Results showed an increase of 25.2% in methane production.

The production efficiency of biogas and methane yield from Cattle Manure (CM) slurry were discussed under the influence of various concentrations of NZVI, ranging from 5 to 20 mg/L. Batch-wise, anaerobic fermentation of CM was conducted at 37 ± 0.3°C, 90 rpm of rotating speed, and 50 days of Hydraulic Retention Time (HRT). This study concludes that the addition of NZVI is favorable for biogas production. The addition of minute amount, amounting to only 5 mg/L, incremented biogas and methane production by 1.44 and 1.38 times, respectively. The best concentration was found to be 20 mg/L which increases biogas and methane volume by 1.45 times and methane production by 1.59 times. The authors mentioned that the addition of these NPs improves the startup of biogas production and hence reduces the lag phase in comparison with control. The optimal NZVI concentration found in this study was further experimented with by the same authors (Abdelsalam et al., 2016).

**FIGURE 5:**
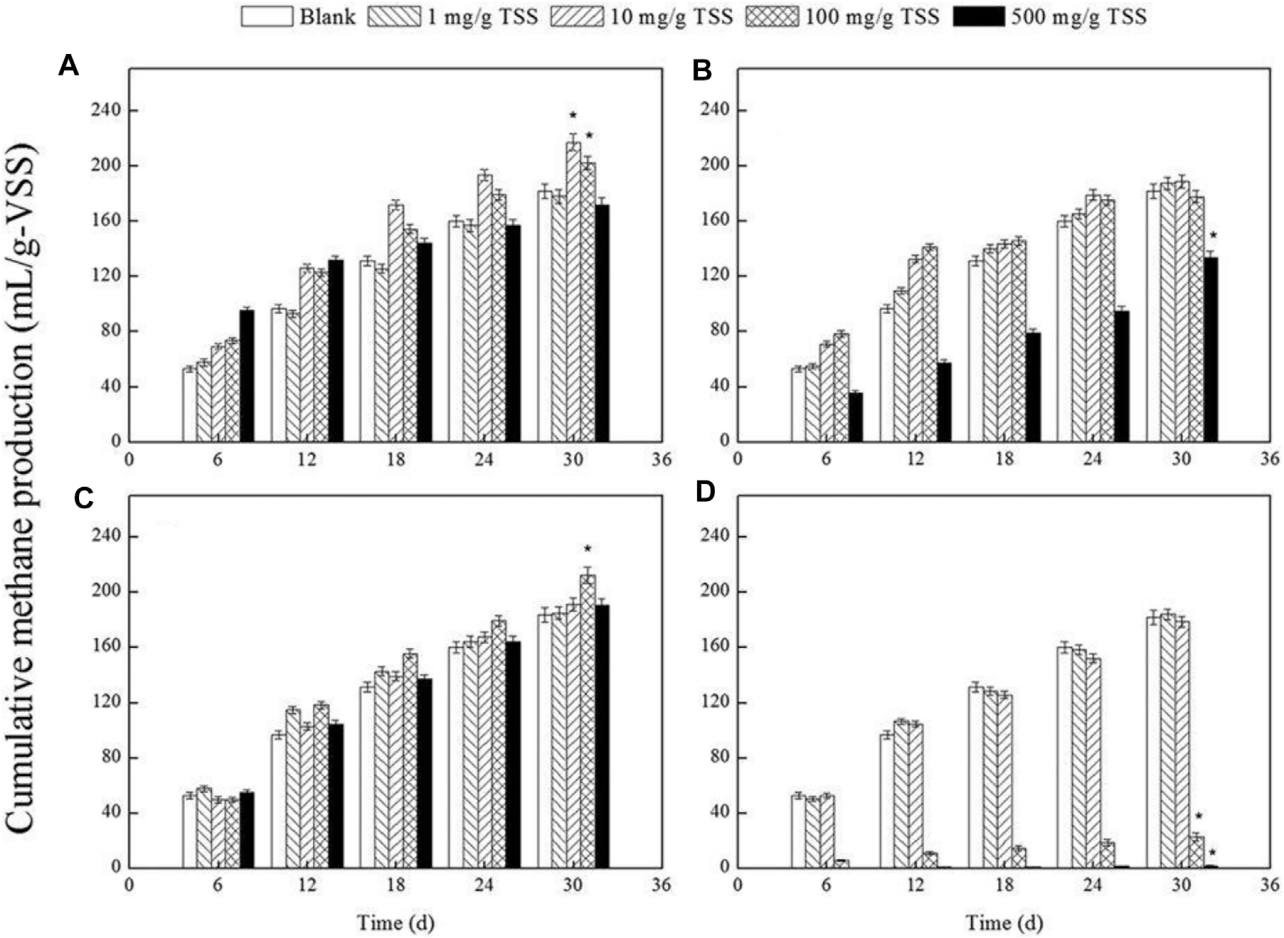
Influence of various concentrations of nZVI **(A)**, Ag NPs **(B)**, Fe_2_ O _3_ NPs **(C)** and MgO NPs **(D)** on cumulative methane production during AD of WAS (Wang et al., 2016).

The influence of NZVI on the AD of WAS was studied by Wang et al. (2016) at concentrations of 1, 10, 100, and 500 mg/g Total Suspended Solids (TSS), respectively. Batch anaerobic digesters were used for the AD with working volume, operating temperature, and mixing rate of 1 L, 35 ± 1°C, and 120 rpm, respectively, for HRT of 30 days. The study indicated that 10 mg/g TSS increased methane production to 120% of the control, whereas other concentrations had no considerable effect, see Figure 5. This is also in agreement with results obtained by Su et al. (Su et al., 2015) and Suanon et al. (Suanon et al., 2016).

In contrast, Amen et al. (2017b) investigated different concentrations of NZVI (50, 100, and 250 mg/L) on anaerobic activated municipal sludge and showed 25% and 62% enhancement in biogas and methane, respectively, by 250 mg/L. In another study conducted by Amen et al. (2017), a novel method of coating NZVI on zeolite and mixing NZVI with zeolite is investigated for improving biochemical methane potential and the lag phase from the AD of anaerobic sludge at 37°C for 14 days of HRT. Zeolite is a mineral compound (a mixture of silica, aluminum, and oxygen). It is a non-cytotoxic mineral having a systematic structure containing channel and pore cavities. The authors worked on the idea that zeolite can trap NZVI inside channels and immobilize the NZVI particles on its surface. Using zeolite as an absorbent carrier for NZVI may be a suitable way to stimulate microorganisms and prevent cell membrane disruptions caused by NZVI. The authors used this method to examine the overall performance of the AD process. It can be observed that till day 8, ICZ caused a lag period, and then from day 9 to day 14, it caused significant biogas enhancement (Amen et al., 2017b). The lag phase is attributed to the time required by anaerobic sludge for the adaptation of ICZ. Results showed that 500 mg/L NZVI and 4 g/L zeolite mixture produced 130.87% increase in cumulative biogas production, whereas NZVI alone (45nm, 1000 mg/L) gave a 105.46% increase in cumulative biogas production. The NZVI coated zeolite (ICZ) with 500, and 1000 mg/L concentrations produced the highest amount of biogas in comparison with other additions and control. Cumulative biogas increase of 149.95% and 286.75% is observed for 500 and 1000 mg/L ICZ, respectively. The study concluded that the higher ICZ concentrations generated more biogas and positively affected the AD process.

**FIGURE 6:**
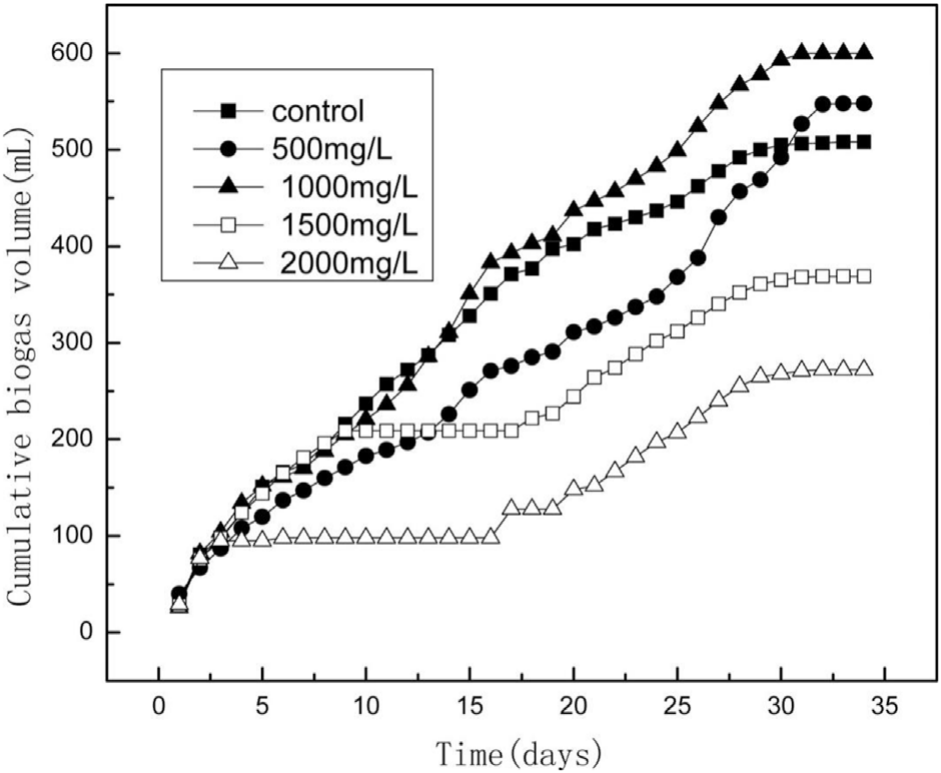
Cumulative biogas production by NZVI (Jia et al., 2017).

The influence of NZVI on wastewater sludge AD was also studied by Jia et al. (2017). The impact of the different concentrations of NZVI (500, 1000, 1500, 2000 mg/L) on wastewater sludge at mesophilic conditions (35°C) for 35 days was investigated. The results showed that the group with 500 mg/L and 1000 mg/L NZVI increased cumulative biogas production by 7.30% and 18.11%, respectively, as shown in Figure 6. The higher concentrations of 1500 mg/L and 2000 mg/L NZVI decreased biogas production by 27.30% and 46.45%, respectively., The higher concentration of NZVI resulted in counter-productive, as observed in other studies (Su et al., 2013; Su et al., 2015; Wang et al., 2016). Therefore, in general, it is critical to find the optimal concentration of the NZVI with the specific waste to achieve the goal, i.e., enhancing biogas generation.

**FIGURE 7:**
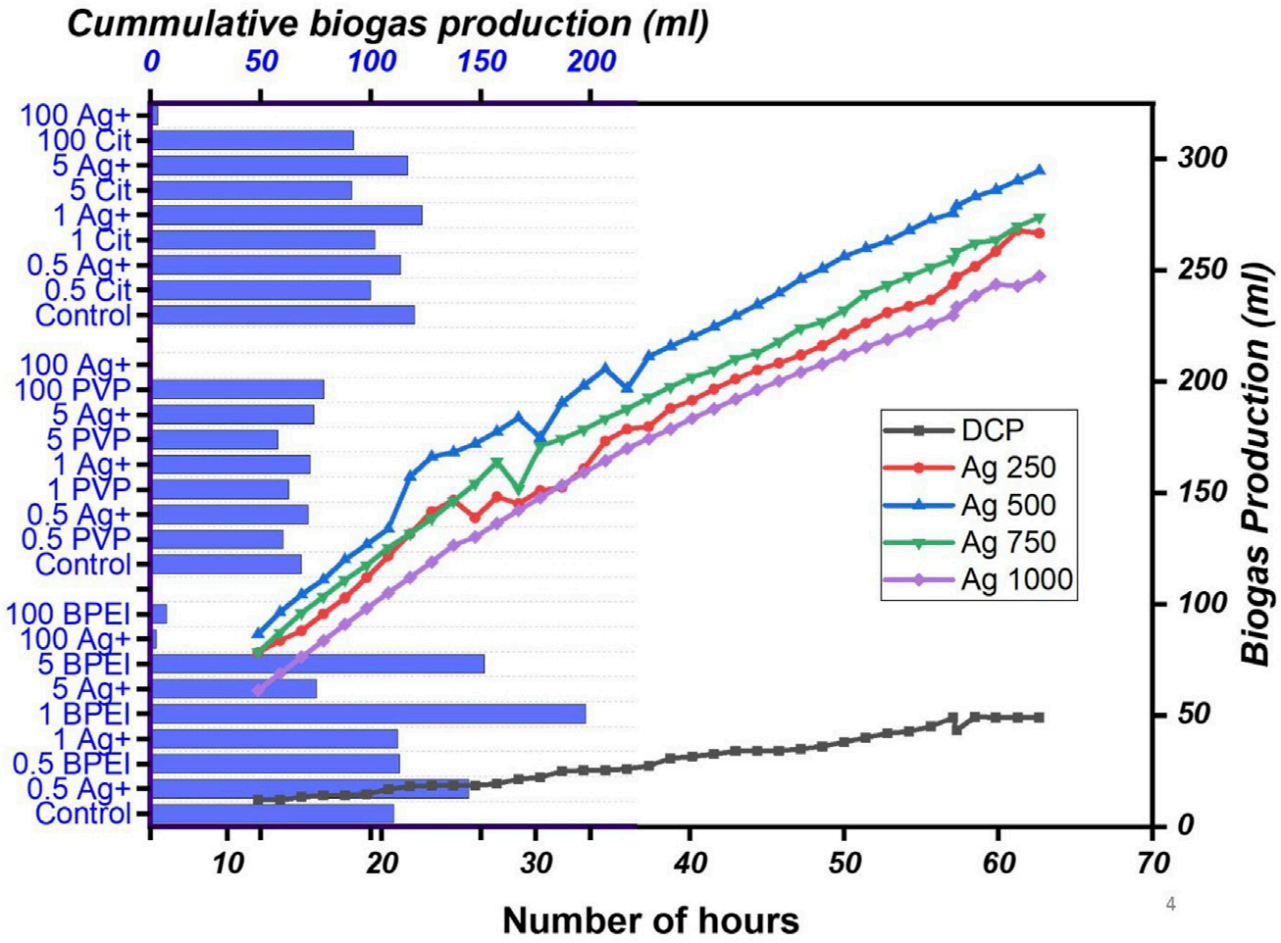
Biogas production for different concentrations of Ag NPs (Ünşar et al., 2016) and Cumulative biogas production (horizontal bar) resulted in different concentrations of Ag NPs or Ag^+^ (Gitipour et al., 2016).

The long and short-term impact of Ag NPs on the AD of waste activated sludge (WAS) was investigated by Ünşar et al. (2016). During the short-term test, Ag NPs did not show any effect on biogas production. However, during the long-term test, high concentrations (150, 250, and 500 mg/g TS) of Ag NPs showed almost 5% inhibition in methane production, see Figure 7. Wang et al. (2016) studied the influence of Ag NPs on the AD of WAS at concentrations of 1, 10, 100, and 500 mg/g TSS, respectively. The study concluded that Ag NPs had no significant effect on biogas production. The 500 mg/g TSS concentration decreases methane production by 73.52%, as shown in Figure 5. Higher concentrations of Ag NPs decrease the biogas yield because they impede the microbes and activities of key enzymes for the AD process. Gitipour et al. (2016) studied the toxicity of cationic Ag NPs on bio-solids from the wastewater treatment plant to examine the antibacterial impacts of different Ag NPs on the AD process and compared to that of Ag^+^. Negatively charged citrate-coated Ag NPs (citrate-Ag NPs), minimally charged polyvinylpyrrolidone coated AgNPs (PVP-Ag NPs), and positively charged branched polyethyleneimine coated AgNPs (BPEI-Ag NPs) were investigated. BPEI-Ag NPs showed a significant increase (almost double the amount) in biogas production than control, as shown in Figure 7. Toxicity examination showed that at lower concentrations of Ag NPs, functional redundancy built within the microbial community resulted in low toxicity. However, at high doses, BPEI-Ag NPs resulted in eminent toxicity compared to PVP-Ag NPs and citrate-Ag NPs.

Abdelsalam et al. (2017a) studied the effects of various concentrations (0.5, 1, and 2 mg/L) of Co. and Ni NPs on the production capability of methane and biogas from the conversion of CM (Abdelsalam et al., 2017a). AD of CM was carried out batch-wise at operating temperature and mixing rate of 37 ± 0.3°C and 90 rpm, respectively, for HRT of 50 days. The study indicated that adding 1 mg/L Co. NPs increases the biogas and methane volume by 1.64 and 1.86 times, respectively. The optimal concentration of Ni NPs was found to be 2 mg/L, which increases biogas and methane volume by 1.74 and 2.01 times, respectively. The authors mentioned that the addition of Ni and Co. NPs improved the startup of biogas production and reduced the lag phase compared to control. Co. and Ni NPs showed increased decomposition of organic matter as more decomposition of Total Solids (TS), and Volatile Solids (VS.) observed at the end of the experiment. Elreedy et al. (2017) also investigated the influence of Ni NPs (60 nm) at much higher concentrations compared to the work in (Abdelsalam et al., 2017a). The Ni NPs concentration in this study was 20, 30, 60, and 100 mg/L on the AD of industrial wastewater containing Mono-Ethylene Glycol (MEG). Results showed that 60 mg/L of Ni NPs produced an increase of 23% in hydrogen production. This result suggested that a higher dose of NPs is required for industrial waste to enhance biogas production. It would be interesting to see that similar waste has been tested for lower NPs concentration for industrial waste, but the authors of this review were unable to find it.

**FIGURE 8:**
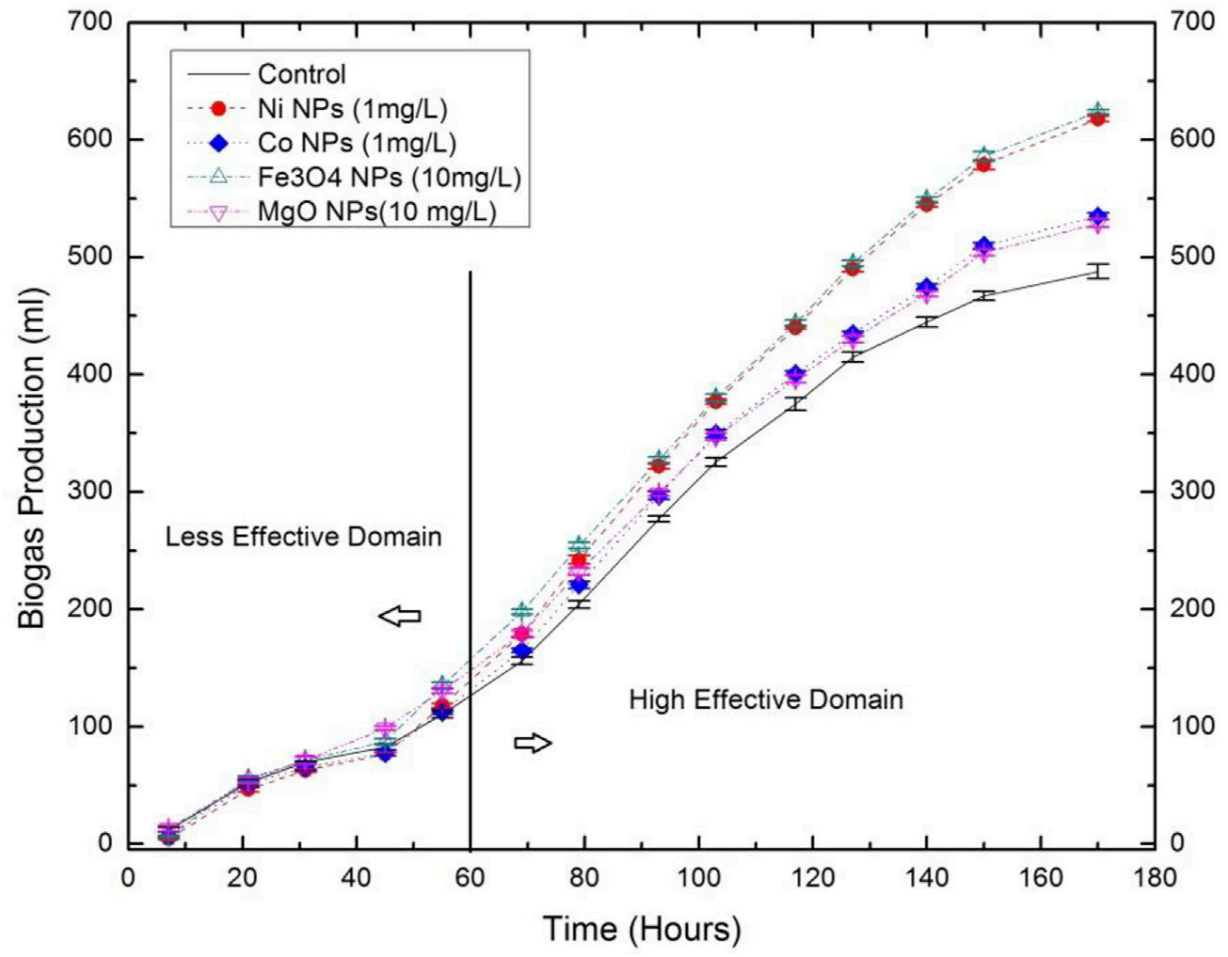
Biogas production influenced by nanoparticles (Zaidi et al., 2018).

Our previous work (Zaidi et al., 2018) explored the influence of Ni and Co. NPs on biogas yield from the AD of green microalgae (*Enteromorpha*), which was the first study to discover the significance of NPs on microalgae. Results indicated that 1 mg/L of Ni and Co. NPs produced 26 and 9% cumulative increase in biogas production. It was observed that during the less effective domain (see Figure 8), NPs revealed no significant result to improve biogas production. However, approximately 60 h of the digestion process, NPs showed the cumulative effect on biogas production. The increase in biogas production was credited to the release of extracellular polymeric compounds (proteins, carbohydrates, and cellulose) after the dissolution of the microalgae cell wall. In order to understand the effectivity of NPs on the AD of microalgal biomass, measurement of soluble indexes such as Chemical Oxygen Demand (COD), reducing sugar, pH and VFA were measured. It was found that COD and VFA increased for the groups with NPs, whereas reducing sugar decreased as NPs stimulated bacteria to consume more sugar during the AD.

An exhaustive list and summary of the reported metal NPs including size, concentration, type of feedstock used, anaerobic temperature, HRT, and their effect on biogas and methane production, is shown in Table 2.

**TABLE 2:** Reported metal NPS and their influence on biogas generation.

NPs type	NPs size	NPs concentration	Feedstock	Temp (^o^C)	HRT	Result	References
NZVI	60–120 nm	0.05 wt%	WAS	32 ± 1	20 days	0.05 and 0.10 wt% NAZI increased methane production by 9.8 and 4.6%, respectively. 0.20 wt% NZVI decreased methane production by 8.8%	Su et al. (2015)
		0.10 wt%					
		0.20 wt%					
	50 nm	0.5 g/L, 1.0 g/L, 2.0 g/L, 4.0 g/L	WAS	35	100 days	Biogas enhanced by the addition of 1 g/L of Fe_3_O_4_ by 21.66%	Xiang et al. (2019)
	50 nm diameter	0.5 g/L, 1 g/L, 2 g/L, 4 g/L	Waste sludge	35.0 ± 2°C	20 days	The optimum dosage for biogas generation was 0.5 g/L of nZVI, promoted the process of hydrolysis-acidification of sludge	Yanru Zhang et al. (2019)
	10 nm	0.04–5000 ppb	Anammox sludge	25.3 ± 1.9°C	310	Ammonium and nitrite utilization rates increased apparently with continuous nZVI addition	Erdim et al. (2019)
		1.25 g/L cNZVI	WWTPS	30	10 days	Reactors dosed with 2.5 and 5.0 g/L cNZVI resulted in equally increased methane production. 1.25 g/L NZVI, both cNZVI, and sNZVI gives 28.3% increase in methane production as compared to respect	Carpenter et al. (2015)
	119–42 nm	2.5 g/L cNZVI					
	123–51 nm	5 g/L cNZVI					
		1.25 g/L sNZVI					
	9 ± 0.3 nm	20 mg/L	CM	37 ± 0.3	40 days	1.5 times and 1.67 times increase in biogas and methane production respectively as compared with control	Abdelsalam et al. (2016)
	50 nm	0.75 and 1.5 g per 500 ml	WWTPS	37 ± 1	12 days	Methane production increases by 1.45 times of the control by 0.75 g dose 70.3% decrease in methane production by 1.5 g dose	Suanon et al. (2016)
	<50 nm	1 mg/g TSS	WAS	35 ± 1	30 days	1 mg/g TSS had no measurable effect. 10 mg/g TSS gives 120% of the control. 100 and 500 mg/g have no considerable effect	Wang et al. (2016)
		10 mg/g TSS					
		100 mg/g TSS					
		500 mg/g TSS					
	7–9 nm	5 mg/L	CM	37 ± 0.3	50 days	5 mg/L NZVI Increase biogas production by 1.44 times and methane production by 1.38 times. 10 mg/L NZVI Increase biogas production by 1.45 times and methane production by 1.53 times. 20 mg/L NZVI Increase biogas production by 1.45 times and methane production by 1.59 times	Abdelsalam et al. (2017b)
		10 mg/L					
		20 mg/L					
	60 nm	50, 100 and 250 mg/L	MSW	37 ± 3	14 days	25.23 and 62.67% increase in biogas and methane production respectively by 250 mg/L	Amen et al. (2017b)
	160 nm	0.1 wt%	WWTPS	37 ± 1	30 days	25.2% increase in methane yield	Suanon et al. (2017)
	45 nm	1000 mg/L	WWTPS	37	14 days	105.46% increase in cumulative biogas production	Amen et al. (2017a)
	50–70 nm	500, 1000, 1500, 2000 mg/L	WWTPS	35	35 days	7.30% increase in biogas production 18.11% increase in biogas yield 27.30% decrease in biogas yield 46.45% decrease in biogas yield	Jia et al. (2017)
	55 nm	56, 560, and 1680 mg/L	Digested sludge	37	14 days	20% decrease in methane production	Yang et al. (2013a)
	20 nm	10 mg/L	Sewage sludge	37	17 days	30.4% increase in biogas production, 40.4% increase in methane production	Su et al. (2013)
	128 nm	10 mg/g TSS	Waste activated sludge	35 ± 1	30 days	Increase 120% of methane production	Wang et al. (2016)
				°C			
	46–60 nm	1500 mg/L	Granular sludge	30 C	-	No toxic effects on the methanogenic activity	
NZVI and zeolite mixture (IMZ)	—	500 mg/L nZVI and 4 g/L zeolite	WWTPS	37	14 days	130.87% increase in cumulative biogas production	Amen et al. (2017a)
NZVI coated zeolite (ICZ)	24.1 μm	500 and 1000 mg/L	WWTPS	37	14 days	149.95% and 286.75% increase in cumulative biogas yield for 500 and 100 mg/L respectively	Amen et al. (2017a)
Ag	20–40 nm	5 mg/g TS	WAS	37	48 days	No substantial decrease in methane yield was detected at 5 and 50 mg Ag per g TS dosages. Dosages of 150, 250, and 500 mg Ag per gTS resulted in more than 5% inhibition. The detected inhibitions as per the investigated dosages are 6.5, 7.8 and 12.1%, respectively	Ünşar et al. (2016)
		50 mg/g TS					
		150 mg/g TS					
		250 mg/g TS					
		500 mg/g TS					
	170 ± 7.9	1 mg/g TSS	WAS	35 ± 1	30 days	1, 10, and 100 mg/g TSS had no measurable effect. 500 mg/g decreased methane production by 73.52%	Wang et al. (2016)
		10 mg/g TSS					
		100 mg/g TSS					
		500 mg/g TSS					
citrate-AgNPs	10–15 nm	0.5 mg/L	WWTPS	37	30 days	No substantial enhancement in biogas	Gitipour et al. (2016)
		1 mg/L					
		5 mg/L					
		100 g/L					
PVP-AgNPs	10–15 nm	0.5 mg/L	WWTPS	37	30 days	No substantial enhancement in biogas	Gitipour et al. (2016)
		1 mg/L					
		5 mg/L					
		100 g/L					
BPEI-AgNPs	10–15 nm	0.5 mg/L	WWTPS	37	30 days	No significant increase in biogas. At 100 mg/L, nearly complete inhibition occurred	Gitipour et al. (2016)
		1 mg/L					
		5 mg/L					
		100 g/L					
Co.	28 ± 0.7 nm	1 mg/L	CM	37 ± 0.3	40 days	1.7 times and 2 times enhancement in biogas and methane production respectively as compared with control	Abdelsalam et al. (2016)
—	<100 nm	0.16 mg/g TSS	Sludge	264 h	37	Co. NPs + MW pretreatment gave 42% cumulative rise in biogas yield	Zaidi et al. (2019b)
—	30–80.9 nm	1.4, 2.7, 5.4 mg/L	Poultry litter	35	69 days Exp. A, 79 days Exp. B	NPs increased CH4 production by 23.8–38.4% compared to poultry litter only AD The highest increase in CH_4_ was observed 29.7% at 5.4 mg/L	Hassanein et al. (2019)
—	<100 nm	1 mg/L	Green algae	37	264 h	For Co. NPs along MW pretreatment enhanced biogas yield by 42.36%	Zaidi et al. (2019b)
—	28 ± 0.7 nm	1 mg/L	Manure slurry	37 ± 0.3°C	50 days	1.64 times and 1.86 times increase in biogas and methane production, respectively as compared with control	Abdelsalam et al. (2017a)
—	17–28 nm	0.5 mg/L	CM	37 ± 0.3	50 days	0.5 mg/L Co. NPs Increase biogas production by 1.36 times and methane production by 1.43 times. 1 mg/L Co. NPs Increase biogas production by 1.64 times and methane production by 1.86 times. 2 mg/L Co. NPs decrease biogas production by 0.95 times and methane production by 0.87 times	Abdelsalam et al. (2017a)
		1 mg/L					
		2 mg/L					
—	100 nm	1 mg/L	Microalgae	37 ± 0.3	7 days	9% increase in biogas production	Zaidi et al. (2018)
—	20 nm	75 mg/L	Cellulose	37 C, 55 C	50 days	Zero or slight toxicity effect on ordinary heterotrophic organisms, ammonia-oxidizing bacteria, and anaerobic bacteria	García et al. (2012)
—	20–40 nm	5, 9, 13 mg/L	SW	35	5 days	The optimum concentration of 9 mg/L was observed with additive 202.46 NL/kg VS., consequently enhanced methane yield by 45%	Yazdani et al. (2019)
—	40–60 nm	9 mg/gVS	Sewage sludge	—	40 days	The 9 mg/gVS increased methane yield by 186% along 2.6 times more VS. removal with respect to the control	Lizama et al. (2019b)
—	40–60 nm	7 mg/gVS+15,000 kJ/kgTS	Sewage sludge	35	30 days	Biogas yield of 190% enhanced while methane of 242.8% increased	Lizama et al. (2019a)
—	30–80.9 nm	15, 50, 100 mg/L	Poultry litter	35	69 days Exp. A, 79 days Exp. B	NPs increased CH4 production by 23.8–38.4% compared to poultry litter only AD Highest increase in CH_4_ was observed 29.1% at 100 mg/L	Hassanein et al. (2019)
	70 nm	2 mg/μg chlorophyll a	Cyanobacte-rial bloom	-	-	promotes flocculation of cyanobacterial biomass	Marsalek et al. (2012)
—	55 ± 11 nm	1680 mg Fe/L (30 mM)	digested sludge	—	—	quick dissolution of Fe NPs NZVI so as to produce hydrogen more	Yang et al. (2013b)
—	<212 μm	1680 mg Fe/L (30 mM)	digested sludge	—	—	By releasing the slow hydrogen from ZVI increases the methane yield higher and sulfate yield gets reduced	Yang et al. (2013b)
—	<50 nm	10 mg/g TSS	waste activated sludge	37	—	In the vicinity of 10 mg/g total suspended solids (TSS) nZVI and 100 mg/g TSS Fe_2_O_3_ NPs enhanced methane yield to 120 and 117% of the control, respectively	Yang et al. (2013b)
—	9 nm	20 mg/L	Raw manure	37 ± 0.3°C	5 days	Methane production was enhanced by 67%	Abdelsalam et al. (2016)
—	0.05 m^2^/g surface area	0.4 g ZVI/g SFW	Food waste	35	30 days	Butyric acid was 30–40% achieved of the VFAs in the acidogenic reactor	Kong et al. (2016)
Ni	17 ± 0.3 nm	2 mg/L	CM	37 ± 0.3	40 days	1.8 times and 2.17 times increase in biogas and methane production, respectively, as compared with control	Abdelsalam et al. (2016)
—	<50 nm	0.004 g/g SS	microalgal biomass	37	15 days	36% enhancement was seen of biomass solubilization	Kavitha et al. (2019)
—	58.3–79.7 nm	1.34 mg/g VS.	Poultry litter	35	69 days Exp. A, 79 days Exp. B	NPs increased CH4 production by 23.8–38.4% compared to poultry litter only AD The highest increase in CH_4_ was observed 38.4% at 12 mg/L	Hassanein et al. (2019)
—	<100 nm	1 mg/L	Green algae	37	264 h	For Ni NPs along with MW pretreatment of enhanced biogas yield by 31.73%	Zaidi et al. (2021b)
—	17 ± 0.3 nm	2 mg/L	Manure slurry	37 ± 0.3°C	50 days	1.74 times and 2.01 times increase in biogas and methane production, respectively, as compared with control	Abdelsalam et al. (2017a)
—	17–28 nm	0.5 mg/L	CM	37 ± 0.3	50 days	0.5 mg/L Ni NPs Increase biogas production by 1.46 times and methane production by 1.49 times. 1 mg/L Ni NPs Increase biogas production by 1.72 times and methane production by 1.96 times. 2 mg/L Ni NPs Increase biogas production by 1.74 times and methane production by 2.01 times	Abdelsalam et al. (2017a)
		1 mg/L					
		2 mg/L					
—	60 nm	20, 30, 60, and 100 mg/L	industrial wastewater containing MEG	55	10 days	60 mg/L dosage caused 23% increase in hydrogen production	Elreedy et al. (2017)
—	100 nm	5 and 10 mg-Ni/kgVS	Sewage sludge	37 ± 1 °C	20 days	increased methane yield up to 10%	Tsapekos et al. (2018)
—	100 nm	1 mg/L	Microalgae	37 ± 0.3	7 days	26% increase in biogas production	Zaidi et al. (2018)
Zn silica nanogel	—	—	Manure	-	56 days	Overall, cumulative gas volumes were decreased by 92.73–95.83%	Sarker et al. (2019)
Mixed NPs	20–21 nm Ag, ZnO, TiO_2_	0.25 mg/g Ag, 2 mg/g TiO_2_, 2.8 mg/g ZnO	Primary activated sludge	35 ± 2°C	300 days	maximum of 73% (control), 71% (ENPs) and 70% (metal salts) methane content in the biogas was observed	Eduok et al. (2017)

Various metal NPs effect on biogas production from different feedstock is presented in this section. NZVI was the most reported one, along with Ni and Co. NPs, which showed an increase in biogas production. On the other hand, Ag, citrate-Ag, PVP-Ag, BPEI-Ag, Au, and Zn silica nanogel showed adverse effects on the biogas production rate, resulting in a dramatic decrease in the amount of biogas produced. This decrease was attributed to the toxicity of the materials.

### Utilization of metal oxide nanoparticles for biogas production

**FIGURE 9:**
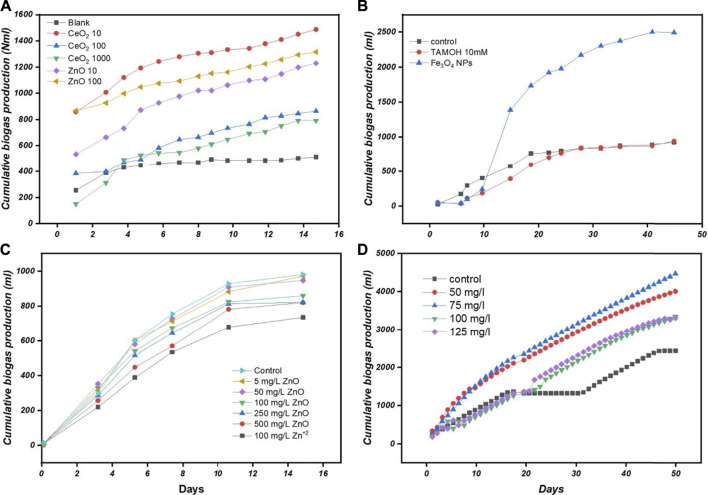
**(A)** Influence of Ce O _2_ and ZnO NPs on biogas production (Nguyen et al., 2015) **(B)** Effect of 100ppm Fe_3_ O _4_ on biogas production (scale bar is 20 nm) (Casals et al., 2014) **(C)** Effect of ZnO ENMs on production after 14 days (Zhang L. et al., 2017) **(D)** Cumulative methane production by Fe_3_ O _4_ NPs (Ali et al., 2017).

The effect of ZnO and CeO_2_ NPs with different concentrations (10, 100, 500, and 1000 mg/L) on anaerobic sludge from an Up-flow Anaerobic Sludge Blanket (UASB) reactor was studied by Nguyen et al. (Nguyen et al., 2015) under mesophilic temperature (30°C) for 40 days. Results showed that all investigated concentrations of ZnO and CeO_2_ NPs produce biogas less than the control except 10 mg/L CeO_2_ NPs sample, which produced only an 11% increase in biogas, as shown in Figure 9. This study remotely suggested that the role of oxides may be limited to use for biogas production; fortunately, this is not the case. The authors performed a bacterial toxicity test to explore the biogas inhibition effect. They found that ZnO NPs are more highly toxic to *Escherichia coli* than CeO_2_ NPs and caused 99% cell death at 100 mg/L and so the same at higher concentrations. The authors attributed the positive effect of 10 mg/L CeO_2_ NPs on the bacterial viability of sludge digestion as their ability to act like free radicals.

The long and short-term inhibition impacts of CuO and CeO_2_ NPs was studied by Ünşar et al. (2016) on the AD of WAS. The AD inhibition effect was observed from 5.8% to 84% when CuO NPs concentration increased from 5 mg/g to 1000 mg/g TS. CeO_2_ NPs with dosages of 150, 250, and 500 mg/g TS enhanced the methane yield to 18.8%, 25.5%, and 9.2%, respectively (Ünşar et al., 2016). Fluorescence *in situ* hybridization (FISH) analysis exposed a decrease in archaea in CuO NPs samples, whereas the abundance of these bacteria was found in CeO_2_ NPs.

Casals et al. (2014) also performed an anaerobic experiment under mesophilic conditions by applying Fe_3_O_4_ NPs (100 ppm) to organic waste for about 2 months. It was concluded that this set of conditions promises an increment in the production of methane and biogas by 234% and 180%, respectively, as shown in Figure 9. In addition, Fe^2+^ was identified as the main contributing factor as it serves to disintegrate waste fabulously in anaerobic conditions. This is probably one of the highest increments of biogas and methane production one can find in the available literature.

In the AD process, metal distribution conversion is another important aspect, as discussed by Suanon et al. (Suanon et al., 2016). The effect was studied by employing Fe_3_O_4_ NPs in an anaerobic batch chamber with mesophilic conditions. The methane production was incremented by 1.5 gm per 500 ml of Fe_3_O_4_ NPs. It was concluded that the presence of Fe_3_O_4_ NPs is favorable for metal stabilization in the digestate as it ultimately results in an improvement in biogas production. However, it promotes the immobilization of phosphorus in digestate. The information mentioned in the paper was not conclusive to support the immobilization hypothesis, and the authors have acknowledged this to suggest further research.

Abdelsalam et al. (2017b) also contributed by studying the effect of Fe_3_O_4_ NPs on biogas production. By employing different concentrations on CM slurry, mixing temperature of 37 ± 0.3°C at an rpm of 90 and HRT of 50 days; biogas and methane production was incremented by 1.66 and 1.96 times, respectively, by adding just 20 mg/L Fe_3_O_4_ NPs as shown in Figure 9.

Wang et al. (2016) studied the influence of MgO and Fe_2_O_3_ NPs on the AD of WAS at concentrations of 1, 10, 100, and 500 mg/g TSS, respectively. The concentration of 100 mg/g TSS of iron oxide NPs gives 117% of the control, whereas other concentrations had no measurable effect on biogas; see Figure 5. MgO NPs had no significant effect on biogas production (Shi et al., 2020). The 500 mg/g TSS concentration inhibited methane production by 1.08%. Higher concentrations of MgO NPs decrease the biogas yield because they impede the microbes and activities of key enzymes for the AD process, see Figure 5.

Li et al. (2017) studied the fate and long-term exposure of CuO, TiO_2,_ and ZnO NPs (50 mg/L) on the AD of Anaerobic Granular Sludge (AGS) for 90 days. The results showed that CuO NPs stopped the methane production on the 39th day. Long-term exposure resulted in inhibited methanogenesis strongly and quickly. The exposure of TiO_2_ NPs lowered the biogas and methane production by 30.70% and 14.01%, respectively. The study suggested that TiO_2_ NPs had an adverse effect on the acidogens and acetogens than methanogens. The effect of TiO_2_ NPs on anaerobic sludge from the UASB reactor was also investigated by Yadav et al. (Yadav et al., 2017). Outcomes of their study indicated a slight biogas inhibition in line with the results obtained by Li et al. (2017).

Syntrophic oxidation of butyrate (intermediates in the transformation of complex organics to methane) was studied by Zhang and Lu (2016) in two different lake sediments. The authors used conductive Fe_3_O_4_ NPs to accelerate the reaction kinetics. Results indicated that methane yield was substantially increased, and the lag phase reduced significantly under the presence of NPs. 25μmol CH_4_/liter was produced from 10 μmol of butyrate addition. The authors performed Direct Interspecies Electron Transfer (DIET) and found that cell-to-cell distance in enrichments amended with NPs was larger than control. They suggested that conductive NPs form cell-nanomaterial-cell networks and facilitate DIET, which contributed to an enhancement in methane.

The response of iron oxide NPs on AGS during AD of beet sugar industrial wastewater was investigated by Ambuchi et al. (2017). Three Plexiglas Expanded Granular Sludge Bed (EGSB) reactors were used under a mesophilic temperature of 36 ± 1°C for an incubation period of 74 days. More biogas was produced during the first 24 h than in the control reactor. The initial increase in biogas production was also observed in another study (Abdelsalam et al., 2017b). Results showed 1.25 times increase in biogas and 28.9% more ml/g-VSS CH_4_ gas. The authors stated that the employment of iron oxide NPs as conduits for electron transfer toward methanogens resulted in biogas enhancement.

A comparative study of Fe_3_O_4_, Co_3_O_4_, NiO, and MoO_3_ micronutrient and NPs with CM slurry in the single and bi-phasic AD at 37 ± 2°C for 20 days was carried out by Juntupally et al. (2017). During a single-phase AD, Fe_3_O_4_ NPs produced 0.16 L/(g VS. reduced) biogas. An increase in biogas production with enhanced methane (70–80%) is reported during single-phase, whereas in bi-phase, AD Fe_2_O_3_ and its corresponding NPs showed a 76% increase (Juntupally et al., 2017). NiO NPs yielded peak biogas of 0.3 L/(g VS. reduced) in the biphasic AD compared to Co_3_O_4_ and MoO_3_ NPs. During single-phasic AD, NiO and Co_3_O_4_ NPs provided the same biogas yield of 0.15 L/(g VS. reduced).

The effect of different concentrations of ZnO NPs (as shown in Figure 9) on VFAs and biogas production during AD of WAS investigated by Lingling Zhang et al. (2017). Results showed that VFA production is inversely correlated to ZnO NPs concentrations. ZnO NPs inhibited the waste sludge hydrolysis-acidification, mainly protein. ZnO NPs’ impact on protein hydrolysis slowed down the VFA accumulation during AD and decreased biogas production, as shown in Figure 9. This action also changed bacterial community structure and was identified to be the main reason for biogas reduction.

Temizel et al. (2017) investigated the influence of ZnO NPs on sanitary landfills for biogas production. They used landfill bioreactors operated at 35°C for 1 year. The results obtained indicated that reactors inoculated with ZnO NPs produced less biogas than the control reactor. The authors mentioned that the release of Zn^2+^ might adversely affect the methanogenic archaea activity, and hence inhibition in biogas yield occurred. Biogas from landfills is being recognized as one potential source for bioenergy production; the authors suggested that the presence of ZnO NPs in a waste matrix of landfills may become a hurdle to its application. The toxic effect of ZnO NPs indicated in this study agrees with Li et al. (2017), who also investigated the effect of ZnO NPs on the AD of AGS and found that methane and biogas yield was suppressed. They mentioned that long-term exposure resulted in inhibited methanogenesis vigorously and quickly.

The effect of bio-compatible Fe_3_O_4_ NPs (10–35 nm) at four different concentrations (50, 75, 100, and 125 mg/L) on the AD of Municipal Solid Waste (MSW) was investigated by Ali et al. (2017) at 37 ± 0.5°C for 60 days of HRT. Results indicated that concentration of 50 and 75 mg/L was found to be more effective in improving the methane production as compared to increased concentrations at 100 and 125 mg/L, see Figure 9. This is in contrast with the results obtained by Abdelsalam et al. (2017b).

In one of our previous studies, the experience of studying green microalgae’s anaerobic digestion (*Enteromorpha*) for biogas production by employing Fe_3_O_4_ and MgO NPs have been promising (Zaidi et al., 2018). A cumulative increase of 28% for 10 mg/L of Fe_3_O_4_ NPs and 8% for 10 mg/L of MgO NPs was noticed. As a controlled sample, an additional effect of NPs approaches zero in the less effective domain. Nevertheless, after observation of 60 h, a substantial effect incrementing biogas production was noticed. The increase in biogas production was credited to the release of extracellular polymeric compounds (proteins, carbohydrates, and cellulose) after the dissolution of the microalgae cell wall. Table 3 comprehensively summarizes the metal oxide NPs and their effect on biogas generation.

**TABLE 3:** Reported metal oxide NPs and their influence on biogas generation.

NPs type	NPs size	NPs concentration	Feedstock	Temp (^o^C)	HRT	Result	Ref
CeO_2_	—	10 mg/L	UASB Reactor Sludge	30 ± 1	40 days	A decrease in biogas was observed. 10 mg/L increase biogas generation by 11%	Nguyen et al. (2015)
		100 mg/L					
		500 mg/L					
		1000 mg/L					
—	15–30 nm	5 mg/g TS	WAS	—	48 days	CeO2 dosages of 150, 250, and 500 mg per gTS enhanced methane generation to 18.8, 25.5, and 9.2%, respectively	Ünşar et al. (2016)
		50 mg/g TS					
		150 mg/g TS					
		250 mg/g TS					
		500 mg/g TS					
—	12 nm	640 mg/L	Cellulose	37, 55	50 days	Toxicity effect, decrease nearly 100% biogas production	García et al. (2012)
—	<25 nm	5, 50, 150 mg/g VSS	GS	35	6	No effect was observed	Ma et al. (2013)
—	50 nm	1500 mg/L	Granular sludge	30	80 h	No toxic effects on the methanogenic activity. Acetoclastic MA is reduced by 80%, while hydrogenotrophic reduced by 82%	Gonzalez-Estrella et al. (2013)
—	192 nm	10 mg/L	Anaerobic sludge	30	40 days	NPs could increase the biogas production by 11%	Hou et al. (2017)
ZnO + Cip	119.7 nm ZnO	0.015, 0.300, and 3.000 mg/g DW ZnO	Sludge	35 ± 2°C	20	Complex inhibition rate of ZnO + C_ip_ decreased by 23.3%	Lin Zhao et al. (2018)
		10,100 mg/kg DW Cip					
ZnO + C_60_	119.7 nm ZnO	0.015, 0.300, and 3.000 mg/g DW ZnO	Sludge	35 ± 2°C	20	ZnO + C_60_ gave an inhibition rise of only 3.9% Complex inhibition rate was 18.5%	Lin Zhao et al. (2018)
	129.5 nm C_60_	100 mg/kg DW C_60_					
ZnO	—	10 mg/L	UASB Reactor Sludge	30 ± 1	40 days	Inhibition in biogas production was observed	Nguyen et al. (2015)
		100 mg/L					
		500 mg/L					
		1000 mg/L					
—	119.7 nm	30 mg/g	Sludge	35 ± 2°C	35 days	The inhibition rate of ZnO was 26.7%	Zhao et al. (2019)
—	119.7 nm	0.015, 0.300, and 3.000 mg/g DW of sludge	Sludge	35 ± 2°C	20	Only ZnO inhibited CH4 yield by 49.5% at 14 h and 15% after 35 days	Lin Zhao et al. (2018)
—	531 nm	0.4 mg/L	seed sludge	35	(SRT = 120 days and HRT = 6 h)	biogas production reduced from 0.36 to 0 L/g COD removal within 40 days	Chen et al. (2019)
—	140 nm	10, 300, 1500 mg/L	waste activated sludge	35	20 days	1 mg/g-TSS of ZnO NPs not affected methane production, 30 and 150 mg/g-TSS of ZnO NPs enhanced 18.3% and 75.1% of inhibition respectively	Mu and Chen, (2011)
—	140 nm	10, 50 mg/g TSS	Aerobic granule	35 ± 1°C	—	No effect noticed	Mu et al. (2012)
—	140 nm	100, 200 mg/g TSS	Aerobic granule	35 ± 1°C	—	Effect of −25.1%,−44.5% were observed	Mu et al. (2012)
—	<100 nm	100 mg nano-ZnO/kg of dry waste	Sanitary Landfills	35 ± 2	1 year	The decrease in biogas production of about 15%	Temizel et al. (2017)
—	<100 nm	6, 30, 150 mg/g TSS	WAS	35	18	6 mg/g, 30 mg/g, 150 mg/g TSS affected methane production by no effect, 23% and 81% repectively	Mu et al. (2011)
—	120–140 nm	42, 210, 1050 mg/L	Mixed primary and excess sludge	35	8 days	Decreased the abundance of methanogenic archaea, inhibition of methane production	Haining Huang et al. (2019)
—	50–70 nm	7.5–480 mg/L	Cattle manure	36	14 days	Inhibition of biogas production up to 74%	Luna-delRisco et al. (2011)
—	10–30 nm	10–1500 mg/L	Granular sludge	30	80 h	highly inhibitory to acetoclastic and hydrogenotrophic methanogens with IC50 values of 87 and 250 mg/L	Gonzalez-Estrella et al. (2013)
—	<100 nm	0.32, 34.5 mg/L	WAS	30	90	In addition to 0.32 mg/L, a slight decrease in methane yield was observed while adding 34.5 mg/L shows complete inhibition in 1 week	Otero-González et al. (2014a)
—	850 nm	10 mg/L 1000 mg/L	Sludge out of UASB reactor	30	40 days	Biogas reduced by 8% using 10 mg/L while 65% reduction is seen when 1000 mg/L added	Hou et al. (2017)
—	90–200 nm	0, 5, 50, 100, 250 and 500 mg/L	WAS	37 ± 1	14 days	Inhibition in biogas and methane was observed with increasing dosages of ZnO NMs. 25% reduction on biogas and 50% reduction on methane production	Lingling Zhang et al. (2017)
—	15 micro.m	120 mg/L	Cattle manure	36	14	18%, 72% reduction in biogas by addition of 120 mg/L, 240 mg/L	Luna-delRisco et al. (2011)
—	<100 nm	50 mg/L	AGS	35 ± 1	90 days	Inhibition effect on biogas and methane yield	Li et al. (2017)
—	200 nm	0, 5, 30, 100 mg/g-TSS	WAS	37 ± 1°C	-	Enzyme activity decreased, thus inhibition reduced in the vicinity of TiO_2_	Lingling Zhang et al. (2019)
CuO	30–50 nm	5 mg/g TS	WAS	—	48 days	CuO NPs inhibited methane from 150 mg CuO per gTS concentration. 150, 250 and 500 mgCuO per gTS dosages resulted in strong inhibition	Ünşar et al. (2016)
		50 mg/g TS					
		150 mg/g TS					
		250 mg/g TS					
		500 mg/g TS					
—	<50 nm	50 mg/L	AGS	35 ± 1	90 days	Inhibition effect on biogas and methane production	Li et al. (2017)
—	30 nm	7.5–480 mg/L	Cattle manure	36	14 days	Inhibition of biogas production up to 96%; 120 mg/L, 240 mg/L show decreasing effect in Biogas production by 19% and 60%	Luna-delRisco et al. (2011)
—	30 nm	15 mg/L	Cattle manure	36	14	30% reduction in biogas in noticed	Luna-delRisco et al. (2011)
—	40 nm	10–1500 mg/L	Granular sludge	30	80 h	Inhibited acetoclastic methanogens with IC_50_ value of 223 mg/L	Gonzalez-Estrella et al. (2013)
—	37 nm	1.4 mg/L	AGS	30	83	Methane yield reduced by 15%	Otero-González et al. (2014b)
Fe_3_O_4_	7 nm	100 ppm	WWTPS	37	60 days	180% increase in biogas production and 234% increase in methane production	Casals et al. (2014)
—	—	10 g/L	waste activated sludge	37 ± 1°C	22 days	Methane yield out of ZVI + Fe3O4 in digester was 68.9% greater than Fe-free digester	Zisheng Zhao et al. (2018a)
—	—	10 g/L	Waste activated sludge	37 ± 1°C	22 days	Fe_3_O_4_ obviously enhanced the sludge’s solubilization, hydrolysis, and acidification	Zisheng Zhao et al. (2018b)
—	20–30 nm	75 mmol	Swine manure	37 ± 0.1°C	38 days	Nano magnetite improved the methane yield by a maximum 6.0%; the maximum methane production may be increased by 47.8% on a daily basis	Junya Zhang et al. (2019)
—	100–150 nm	50 mg/g	Lignocellulos-se degradation	37%	60 days	HA enhanced by 54% Fe_3_O_4_ were observed more random after solid-state fermentation	Danlian Huang et al. (2019)
—	7 nm	100 mg/L	Wastewater sludge	—	480 days	Short term exposure of AgNPs evidently decreased nitrogen removal Long-term exposure to AgNPs had no rigorous effects	Juan Huang et al. (2019)
—	7.2 nm	120 ppm (12 mg/g VS.)	Rice straw	37	15 days	2% NaOH with 120 ppm NPs increase CH_4_ production nanoparticles increased methane yield by 129%.	Khalid et al. (2019)
—	94–3400 nm	15, 50, 100 mg/L	Poultry litter	35	69 days Exp. A, 79 days Exp. B	NPs increased CH4 production by 23.8–38.4% compared to poultry litter only AD The highest increase in CH_4_ was observed 27.5% at 15 mg/L	Hassanein et al. (2019)
	100 nm	0.162 mg/g VS.	canola straw and banana waste plant with buffalo dung	37 ± 0.1	40 days	Maximum methane yield of 256 mLCH_4_/gVS and 202.3 mLCH4/gVS at a dosage of 0.81 & 0.5 mg for CS and BPW	Noonari et al. (2019)
	20 nm diameter	0.5 g/L, 1 g/L, 2 g/L, 4 g/L	Waste sludge	35.0 ± 2°C	20 days	The optimum dosage for biogas generation was 1 g/L of Fe_3_O_4_	Yanru Zhang et al. (2019)
	7 ± 0.2 nm	20 mg/L	CM	37 ± 0.3	40 days	1.7 times and 2.16 times increase in biogas and methane production respectively as compared with control	Abdelsalam et al. (2016)
	1212.6 ± 109.4 nm	1.43–17.1 mg/g MLSS	synthetic wastewater	25	57 days	Fe_3_O_4_ NPs at 5–60 mg/L showed no substantial effect on N removal, moreover on COD removal with a slight -decrease	Ma et al. (2017)
	20 nm	0.75 and 1.5 g per 500 ml	WWTPS	37 ± 1	12 days	Methane production increases by 1.25 times of the control by 0.75 g dose 0.9 times increase in methane production by 1.5 g dose	Suanon et al. (2016)
	-	10 Mm	lake sediments	-	40 days	CH_4_ production was about 60–90% larger	Zhang and Lu, (2016)
	7–9 nm	5 mg/L	CM	37 ± 0.3	50 days	5 mg/L Fe3O4 NPs Increase biogas production by 1.63 times and methane production by 1.82 times. 10 mg/L Fe3O4 NPs Increase biogas production by 1.64 times and methane production by 1.90 times. 20 mg/L Fe3O4 NPs Increase biogas production by 1.66 times and methane production by 1.96 times. 66% increase in biogas production, 96% increase in methane production	Abdelsalam et al. (2017a), Abdelsalam et al. (2017b)
		10 mg/L					
		20 mg/L					
	10–35 nm	50, 75, 100, 125 mg/L	MSW	37 ± 0.5	60 days	The concentration of NPs 50 and 75 mg/L was found to be more effective in improving the methane production as compared to increased concentrations at 100 and 125 mg/L	Ali et al. (2017)
	7 nm	100 mg/L	crystalline cellulose	37	60 days	180% increase in biogas production, 8% increase in methane production	Casals et al. (2014)
	15–22 nm	50–125 mg/L	Municipal solid waste	37	60 days	Up to 117% increase in methane production	Ali et al. (2017)
	<100 nm	10 mg/L	CM	37 ± 2	20 days	Increase in biogas production with enhanced methane (70–80%)	Juntupally et al. (2017)
	20 nm	750 mg/L	BSIWW	36 ± 1	74 days	1.25 times increase in biogas. 28.9% more ml/g-VSS CH4 gas	Ambuchi et al. (2017)
	<100 nm	10 mg/L	Microalgae	37 ± 1	7 days	26% increase in biogas production	Zaidi et al. (2018)
Fe_2_O_3_	<30 nm	1 mg/g TSS	WAS	35 ± 1	30 days	1, 10 and 500 mg/g TSS had no influence. 100 mg/g TSS gives 117% of the control	Wang et al. (2016)
		10 mg/g TSS					
		100 mg/g TSS					
		500 mg/g TSS					
	20 nm	0.5 g/L, 1.0 g/L, 2.0 g/L, 4.0 g/L	WAS	35	100 days	Biogas enhanced by the addition of 0.5 g/L of Fe_3_O_4_ by 24.44%	Xiang et al. (2019)
	20–40 nm	20 mg/L	Cattle Manure	38	30 days	production of biogas and CH_4_ was 336.25 and 192.31 ml/gVS, respectively, at max Fe_2_O_3_ NPs improved anaerobic digestion, resulting in higher production of methane	Farghali et al. (2019)
		100 mg/L					
	140 ± 30 nm	500 mg/g TS	Waste activated sludge	25	48 days	Methane production was decreased by 289%	Kökdemir Ünşar and Perendeci, (2018)
	-	750 mg/L	Granular sludge	36	84, 96 h	Increase 38% of methane production	Ambuchi et al. (2016)
	40 nm	1500 mg/L	Granular sludge	30	80 h	No toxic effects on the methanogenic activity	Gonzalez-Estrella et al. (2013)
TiO_2_	<100 nm	100 mg/L	UASB Reactor Sludge	37	15 days	No substantial effect on biogas production	Yadav et al. (2017)
	4–8 nm	0, 500, 1000, 1500, 2000 mg/L	wastewater, waste sludge	35 ± 1°C	28 days	methane production increased by an average of 14.9%	Cervantes-Avilés et al. (2018)
	25 nm	50 mg/L	AGS	35 ± 1	90 days	Decreased biogas and methane yield by 30.70% and 14.01%, respectively	Li et al. (2017)
	25 nm	1500 mg/L	Granular sludge	30	80 h	No toxic effects on the methanogenic activity	Gonzalez-Estrella et al. (2013)
	150–170 nm	42, 210, 1050 mg/L	Mixed primary and excess sludge	35	8 days	No measurable impact on methane production	Zheng et al. (2015)
	7.5 nm	840 mg/L	Cellulose	37, 55	50 days	No effects	García et al. (2012)
	<25 nm	6, 30, 150 mg/g TSS	WAS	35	48 h	No effect was seen	Mu et al. (2011)
	185 nm	150 mg/g TSS	WAS	35	105	No effect was observed	Chen et al. (2014)
MgO	<50 nm	1 mg/g TSS	Waste activated sludge	35 ± 1	30 days	1, 10 and 100 mg/g TSS had no measurable effect. 500 mg/g decreased methane production by 108%	Wang et al. (2016)
		10 mg/g TSS					
		100 mg/g TSS					
		500 mg/g TSS					
—	<100 nm	10 mg/L	Microalgae	37 ± 1	7 days	8% biogas enhancement	Zaidi et al. (2018)
—	<50 nm	500 mg/g TSS	WAS	35 ± 1°C	2 days	MgO NPs created up to lower levels of methane yield by 1.08% than of the control	Wang et al. (2016)
Co_3_O_4_	<100 nm	10 mg/L	CM	37 ± 2	20 days	Increase in biogas production with enhanced methane (70–80%)	Juntupally et al. (2017)
NiO	<100 nm	10 mg/L	CM	37 ± 2	20 days	Increase in biogas production with enhanced methane (70–80%)	Juntupally et al. (2017)
—	—	20 mg/L	Sludge from wastewater	50	7–14 days	30% increment compared to the control, which can be elaborated by the prevalence of acetic acid production	Elreedy et al. (2019)
Ni-Ferrite and Ni-Co-Ferrite	∼11 nm	20, 70 and 130 mg/L of both	Cow manure	(15°C)	35-days	Ni-Ferrite NPs achieved biogas enhancements of 30.8%, 28.5%, and 17.9% at concentrations of 20, 70 and 130 mg/L, respectively	Abdallah et al. (2019)
Ni/Co. oxide to palm oil mill effluent	∼14 nm (NiO)	0.41–0.69 g/L (test) and 0.66 g/L (control)	palm oil mill effluent	35°C	110 h	H_2_ gas production was enhanced by 37%	Mishra et al. (2019)
	∼16.79 nm for CoO						
Fe/GAC	50 nm	1000 mg/L	tetracycline wastewater	51 days	35 ± 1 C	The biogas production and methane content were enhanced by 21.2% and 26.9%	Zhang et al. (2018)
Mn_2_O_3_	-	1500 mg/L	Granular sludge	30	80 h	No toxic effects on the methanogenic activity	Gonzalez-Estrella et al. (2013)
SiO_2_	10–20 nm	1500 mg/L	Granular sludge	30	80 h	No toxic effects on the methanogenic activity	Gonzalez-Estrella et al. (2013)
—	10–20 nm	630,150 mg/g TSS	WAS	35	Different time	No significant effect is noticed	Mu et al. (2011)
Al_2_O_3_	<50 nm	1500 mg/L	Granular sludge	30	80 h	No toxic effects on the methanogenic activity	Gonzalez-Estrella et al. (2013)
—	270 ± 10 nm	250 mg Al_2_O_3_/g TS	waste activated sludge	—	—	14.8% increase in methane production	Kökdemir Ünşar and Perendeci, (2018)
—	<50 nm	6, 30, 150 mg/g TSS	WAS	35	Several fermentation time	No effect was observed	Mu et al. (2011)
ɤ-Al2O3	20–50 nm	100 g/L	Granular sludge	27	12 h	Much reduction in methane yield up to 60%	Alvarez and Cervantes, (2012)
Fe_2_NiO_4_	—	100 mg Ni^2+^/L	Wastewater	30	7 days	positive effect of Fe_2_NiO_4_ nanoparticles on AD activity	Chen et al. (2018)
Fe_2_NiO_4_Zn	—	100 mg Ni2+/L	Wastewater	30	7 days	negative effect of Fe_4_NiO_4_Zn nanoparticles on AD activity	Chen et al. (2018)
MoO_3_	<100 nm	10 mg/L	CM	37 ± 2	20 days	Increase in biogas production with enhanced methane (70–80%)	Juntupally et al. (2017)

This section discussed the addition of different metal oxide NPs during the AD for biogas production. Fe_2_O_3_, Fe_3_O_4_, Co_3_O_4_, NiO, MoO_3_ showed an increase in biogas production. On the other hand, CeO_2_ showed mixed effects depending on their concentration in the reactor as well as the digestion time. The addition of nano-iron oxide (Fe_3_O_4_) enhanced methane production by 234% due to the presence of the non-toxic Fe3^+^ and Fe2^+^ ions. ZnO, CuO, TiO_2_, MgO, MnO_2_ showed a decrease or no change in biogas production rate (Mishra et al., 2018).

### Nano-scaled Nb-based compounds in biogas

The functionality of Nb-based compounds (NbO_2_, Nb_3.49_N_4.56_O_0.44_, and NbN) with various concentrations (7.5, 15, 30, 60, and 120 mg/L) at mesophilic condition (36 ± 1°C) in the AD of dairy manure was investigated by Taihong Zhang et al. (2017). This is the first study discussing the application of these compounds for AD. The results showed that Nb-based compounds worked as efficient catalysts in the AD process. They improve the fermentation condition and stimulate the bacterial activity inside the digester. The cumulative biogas production by NbO_2_, Nb_3.49_N_4.56_O_0.44_, and NbN produced was 522.7, 437.1, and 455.7 ml/g VS., respectively (Zhang T. et al., 2017). Table 4 summarizes reported Nb-based compounds and their effect on biogas production.

**TABLE 4:** Reported nano-scale Nb-based compounds and their influence on biogas generation.

NPs type	NPs size (nm)	NPs concentration	Feedstock	Temperature (^o^C)	HRT	Result	References
NbO_2_	200	7.5, 15, 30, 60, and 120 mg/L	DM	36 ± 1	35 days	1.3 times increase in biogas by 60 mg/L concentration	Lingling Zhang et al. (2017)
Nb_3.49_N_4.56_O_0.44_	500	7.5, 15, 30, 60, and 120 mg/L	DM	36 ± 1	35 days	1.1 times increase in biogas by 15 mg/L concentration	Lingling Zhang et al. (2017)
NBN	100	7.5, 15, 30, 60, and 120 mg/L	DM	36 ± 1	35 days	60 mg/L NbN improved cumulative biogas by 1.1 times	Lingling Zhang et al. (2017)

### Nano-scaled transition metal carbides for biogas enhancement

**FIGURE 10:**
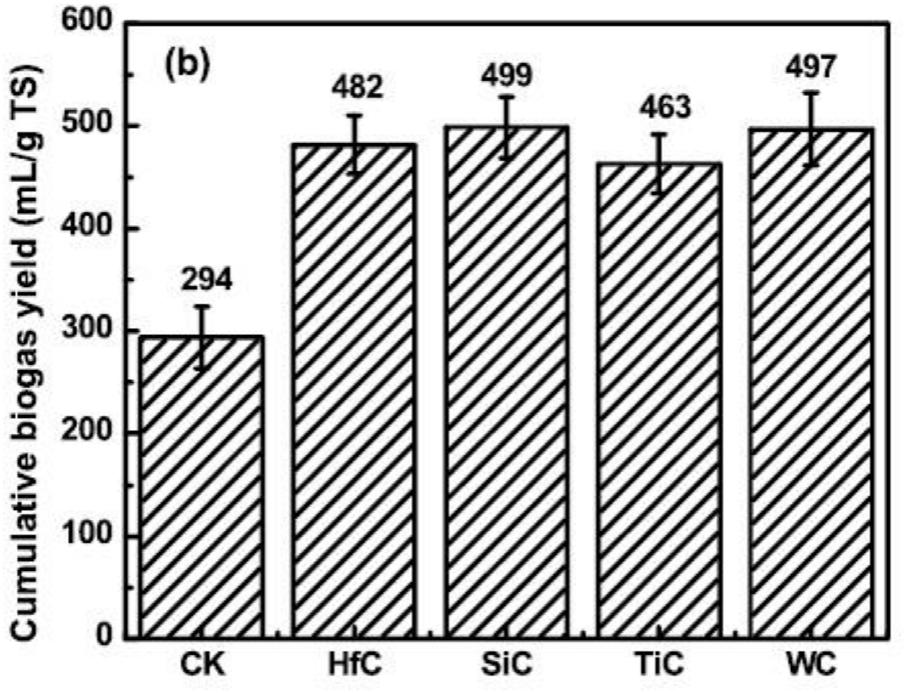
Cumulative biogas yield by nano-scale transition metal carbides (Li et al., 2018).

The effect of nano-scale transition metal carbides (HfC, SiC, TiC, and WC) at a concentration of 0.25 wt% on the AD of CM was investigated by Li et al. (2018) batch-wise under mesophilic temperature. The experiments were performed in triplicates and average data was presented. Results showed that all these four carbides worked as accelerants in the AD process. HfC, SiC, TiC and WC increased biogas production by 63.9, 69.7, 57.5 and 69%, respectively, as compared to control check (CK), see Figure 10. We found that this is the first and maybe the only report on using metal carbides to inoculate in AD digesters. Table 5 summarizes nano-scale transition metal carbides and their influence on biogas generation.

**TABLE 5:** Reported nano-scale transition metal carbides their influence on biogas generation

NPs type	NPs size (nm)	NPs concentration	Feedstock	Temperature (^o^C)	HRT	Result	References
HfC	300	0.025 wt%	CM	37 ± 1	35 days	63.9%increase in cumulative biogas production	Li et al. (2018)
SiC	40	0.025 wt%	CM	37 ± 1	35 days	69.7% increase in cumulative biogas production	Li et al. (2018)
TiC	70	0.025 wt%	CM	37 ± 1	35 days	57.5% increase in cumulative biogas production	Li et al. (2018)
WC	400	0.025 wt%	CM	37 ± 1	35 days	69% increase in cumulative biogas production	Li et al. (2018)

### Utilization of carbon and carbon-based nanomaterials for biogas

**FIGURE 11:**
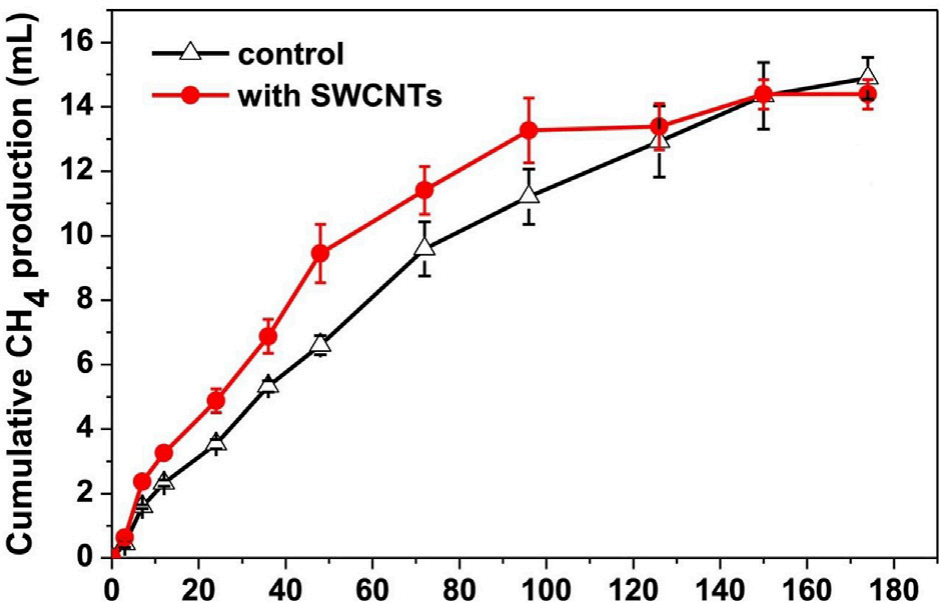
The influence of SWCNTs on methane production in Hours (Li et al., 2015).

The one and the only study discussing the influence of Single-Walled Carbon Nanotubes (SWCNTs) on AD of AGS, with average diameters of 1–2 nm and length of 5–20 nm at a concentration of 1000 mg/L, under mesophilic conditions (35°C) for 8 days was examined by Li et al. (2015). SWCNTs did not reflect any significant enhancement in biogas and methane generation, see Figure 11. In the presence of 1000 mg/L SWCNTs, the volume of generated CH4 was significantly larger (*p* < 0.05) than that in the control reactor for the initial 48 h. However, it slowly decreased and ended at almost the same or little lower cumulative production as control, showing no effect. The authors attributed this zero effect of SWCNTs as a decrement in cytotoxicity of sludge by nanotubes. The addition of SWCNTs in the AD system produced a more Extracellular Polymeric Substance (EPS) which prevented SWCNTs from reaching cells and hence resulted in limited to no effect on biogas yield.

Impact of Multi-Walled Carbon Nanotubes (MWCNTs) with the length of 1–10 μm, outer and inner diameters of 5–20 nm and 2–6 nm, respectively, were investigated on UASB microflora by Yadav et al. (Yadav et al., 2016). It was observed from SEM and fluorescent microscopy images that MWCNTs damaged acidogenic and acetogenic microbial cells, which caused an increase in EPS proteins, DNA, and carbohydrates. According to the authors, this microbial cell damage is the possible reason for low VFAs generation and biogas yield. The 1 mg/L and 100 mg/L concentration of MWCNTs caused 21% and 54% inhibition in biogas as compared to control.

In contrast, Zhang and Lu (2016) found an enhancement in biogas production with conductive MWCNTs (diameter: 10–20 nm, length: 10–30 mm) by syntrophic oxidation of butyrate in two different lake sediments. The CH_4_ production rate in the presence of MWCNTs was almost 50% greater than the control. The results showed that the electric conductivity of the added MWCNTs facilitated the syntrophic oxidation of butyrate and had a stimulatory effect on microorganisms. Microscopic observation showed that abundant aggregates formed in lake enrichments under the presence of MWCNTs. The microbial aggregates in control were in close physical proximity whereas, in MWCNTs samples, dark areas within aggregates filled with nanotubes. This showed that greater intercellular distances existed on average, which form cell-nanotube-cell networks and facilitate DIET, which contributed to an increase in methane yield.

In another study, Ambuchi et al. (2017) investigated the response of MWCNTs (10–20 nm outer diameter) on AGS during AD of beet sugar industrial wastewater. An increase in biogas (1.09 times than control) and methane production (12.6% more ml/g-VSS CH_4_ gas than control) was observed. Summarized results reported that carbon nanotubes influence on biogas generation is shown in Table 6.

**TABLE 6:** Reported carbon nanotubes and their influence on biogas generation.

NPs type	NPs size	NPs concentration	Feedstock	Temperature (^o^C)	HRT	Result	References
SWCNT	Diameter 1–2 nm, length 5–20 nm	1000 mg/L	AGS	35	8 days	No effect	Li et al. (2015)
	1–2 nm diameter, 5–30 μm length	10000 mg/L	Glucose	55	20 days	CH4 production rate increased by 92%	Yan et al. (2017)
MWCNT	length 1–10 μm, outer diameter 5–20 nm and inner diameter 2–6 nm	1 and 100 mg/L	UASB Reactor Sludge	37 ± 1	15 days	21% reduction in the test sample with 1 mg/L MWCNTs and 54% in the test sample with 100 mg/L as compared to control	Yadav et al. (2016)
—	2–20 μm length, 20–30 nm diameter	50 mg/kg, 500 mg/kg	Sheep manure	35	45	presence of 500 mg/kg multiwall carbon nanotubes increased the daily and accumulative production of methane by 46.8 and 33.6%	Hao et al. (2019)
—	10–20 nm in diameter and 10–30 mm in length	0.5% (w/v)	lake sediments	—	40 days	CH4 generation rate was almost 50% larger	Zhang and Lu, (2016)
	10–20 nm outer diameter	1500 mg/L	BSIWW	36 ± 1	74 days	1.09 times increase in biogas. 12.6% more ml/g-VSS CH4 gas	Ambuchi et al. (2017)
	-	1500 mg/L	Granular sludge	36	96 h	Increase 43% of methane production	Ambuchi et al. (2016)
Graphene	4–20 nm	0.5–2 g/L	Ethanol	35	—	Increase 25% in methane yield and 19.5% in biogas production rate	Lin et al. (2017)
—	—	30–120 mg/L	Glucose	35	55 days	Up to 51.4% increase in methane production rate	Tian et al. (2017)
Fullerene (C_60_)	—	50,000 ng/kg of biomass	Waste water sludge	Ambient Temp	89, 154 days	No effect observed	Nyberg et al. (2008)
—	40–60 nm	50 mg/kg, 500 mg/kg	Livestock Sheep manure	35	45	The highest value of daily methane yield was 3.269 ml/g VS., is evident in the 500 mg/kg C_60_ treatment	Hao et al. (2019)
—	129.5 nm	100 mg/kg DW	Sludge	35 ± 2°C	20	No significant change in methane yield, hence failed to alter	Lin Zhao et al. (2018)

### Nanowires, nano composites and nano-ash augmentation for biogas

#### Nanowires

The Octahedral Molecular Sieve (OMS-2) is a form of manganese dioxide that holds distinctive features like mixed-valence of manganese, acidic sites and has wide applications. The effect of synthesized OMS-2 NPs (diameter of nanofibers of about 10–20 nm and lengths of about 100–500 nm) on Sludge from the sewage treatment plant at concentrations of 0.025, 0.25, and 2.50 g/L was investigated by Pan et al. (2015). The addition of 0.025 g/L OMS-2 NPs resulted in an 11% enhancement in biogas production. The investigation of microbial metabolism revealed an increase in microbial metabolic level and enhanced microbial diversity. OMS-2 NPs also increased the quantities of acetogenic bacteria and Archaea and promoted acetogenesis and methanogenesis.

Lupitskyy et al. (2018) studied the influence of zinc oxide nanowires at a concentration of 1 g/L on the AD of AGS. According to the author, the use of ZnO nanowires as inorganic reactive absorbents can help in reducing the sulfur-containing compounds in wastewater and improve biogas production. The experiment was carried out for three feeding cycles. Sulfates were added at the beginning of each feeding cycle. Results showed that nanowires reduced the sulfide toxicity during AD as no methanogenic activity and biogas inhibition were observed (Lupitskyy et al., 2018). The summary of the reported nanowire and its influence on biogas generation is shown in Table 7.

**TABLE 7:** The reported nanowire, nano-composite, nano-ash, and their influence on biogas generation.

NPs type	NPs size	NPs concentration	Feedstock	Temperature (^o^C)	HRT	Result	References
OMS-2	Dia of nanofibers is about 10–20 nm, lengths are about 100–500 nm	0.025, 0.25, and 2.50 g/L	WWTPS	35	189 days	11%increase in biogas production	Pan et al. (2015)
ZnO Nanowire	-	1 g/L	AGS	35	60 h	No argumentative effect on the methanogenic activity was found	Lupitskyy et al. (2018)
Ni-Gr Nano -composite	23 nm	10, 20, 30, 60 and 100 mg/L	industrial wastewater containing mono-ethylene glycol (MEG)	55	240 h	60 mg/L dosage caused 105% increase in hydrogen production	Elreedy et al. (2017)
Micro Nano Fly Ash	0.4–10,000 nm	3 g/g VS.	MSW	35	90 days	Biogas enhancement by 2.9 times	Lo et al. (2012)
Micro Nano Bottom Ash	0.4–10,000 nm	36 g/g VS.	MSW	35	90 days	Biogas enhancement by 3.5 times	Lo et al. (2012)
Ni-Co-Ferrite	—	0–140 mg/L	Cow Manure	38	35 days	32.8% increase in biogas production	Mansour et al. (2020)
Zinc ferrite	6.22 nm	500 mg/L	Cattle manure	40	50 days	185.3% increase in biogas production	Hassaneen et al. (2020)

#### Nano-composites

**FIGURE 12:**
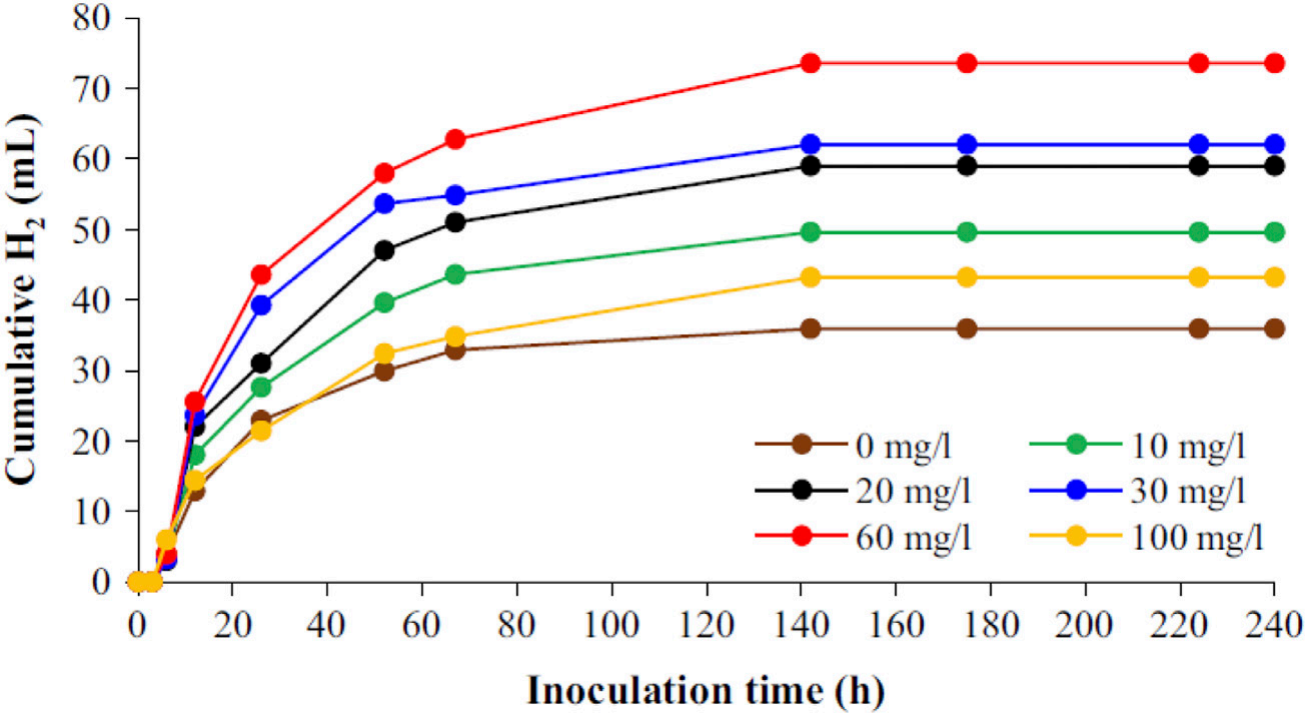
Cumulative hydrogen production at different concentrations of Ni-Gr NC (Elreedy et al., 2017).

The effect of Ni-graphene nano-composite (Ni-Gr-NC) as a supplement to an AD of industrial wastewater containing MEG to enhance biohydrogen production was studied by Elreedy et al. (2017). The authors used the unique properties of Ni-based NPs as Ni ion suppliers and graphene as support materials. This is the first study with Ni-Gr-NC addition to the AD process. The results showed that 60 mg/L dosages caused a 105% increase in hydrogen production from other concentrations. The maximum specific hydrogen production obtained by Ni-Gr-NC (60 mg/L dose) was 294.24 ± 12.06 ml/L, see Figure 12. The hydrogenase enzyme activity affected by Ni ions in the presence of graphene resulted in an enhanced hydrogen yield. The summary of the reported nano-composites and their influence on biogas generation is shown in Table 7.

Mansour et al. (2020) studied the effect of Ni-Co-Ferrite on biogas production and reported that these nano additives increase biogas production by about 30%. In another study, Hassaneen et al. (2020) proposed the use of a novel nanocomposite (based on metal enzyme cofactors, highly conductive carbon materials, and DIET activators) and tested different formulations for the enhancement of biogas production. Methane production was observed to boost by 185.3% using Zn ferrite.

#### Nano-ash

The influences of micro-nano fly and bottom ash attained from MSW incinerator on the AD of MSW were investigated by Lo et al. (2012) at mesophilic conditions (35°C) for 90 days. The concentrations used for micro-nano fly ash was 0.12, 3, 6, 18, and 30 g/g VS. whereas micro-nano bottom ash was used at the concentration of 0.6, 12, 36, 60, and 120 g/g VS Results indicated that micro-nano fly and bottom ash produced a significant enhancement in biogas generation. The inoculation of 36 g/g VS. bottom ash produced the highest amount of biogas production among all dosages, as shown in Figure 12. The authors mentioned that the presence of various compounds (Al_2_O_3_, ZnS, CaCO_3_, CaMg(CO_3_)_2_, Ca_3_SiO_5_, Ca(OH)_2_, PbO, SiO_2,_ and Ca_2_SiO_4_) inside fly and bottom ash increased biogas production. The compounds present in the form of nano-substances supplied additional habitats for the microorganism. The summary of the reported nanoash and its influence on biogas generation is shown in Table 7.

## Key challenges and way forward to nanomaterials augmentation in biogas production

Nanomaterials as additives to biomass were widely studied for biogas production enhancement, especially in the last decade. Unfortunately, their use may not always enhance biogas production, depending on many factors such as the size of nanomaterials, their concentration, and the type of substrate used. However, it is observed that nanomaterials used in the mixture tend to produce a much better effect on biogas production than separately used. Using different nanomaterials as a mixture and studying their interactions with different substrates could be a leading field research area in the years to come.

Furthermore, the environmental impact of NMs application with biomass for biogas production has not been discussed thoroughly, and climate concerns remain high for spent biomass with NMs. One of the significant challenges that need to be addressed urgently is that after utilizing NMs in AD, how to track them, and what would be the best methodology for dumping the waste and biomass that contains NMs? There is a possibility that spent biomass with a high concentration of NMs may prove beneficial for soil and help maintain a nutrient level in the soil. On the other hand, these nanomaterials can increase the toxicity of the area and can also mix with underground water. These aspects have to be answered in future studies. Moreover, multiple studies can be found on the feasibility and financial aspect of NMs application in biogas production throughout the literature. However, studies related to NMs in biomass applications’ environmental analysis and life cycle assessment are quite rare, which needs attention in future studies. The review and analysis of the available literature conducted in this study, the future direction, research area, and themes are depicted in Figure 3B. Currently, the most active countries working on nanotechnology-based biogas production as per citation record (minimum 100 documents and 100 citations) are presented in Figure 4. In addition, future guidelines may comprise the following:

1. In order to avoid the toxicity of the presently spent nanomaterials, causing an inhibitory effect on anaerobic bacteria, bioactive nanomaterials can be used for process improvement.
2. Recollecting spent nanomaterials at the end of the process remained a significant drawback for the environment and sustainability of their utilization in biogas or related applications. Avoiding the leak of nanomaterials in the natural resources and designing processes that limit this to happen should be the top priority for the implementation for large-scale production.
3. Optimization of nanomaterials for a wide range of sizes, doses, and shapes can be carried out to get the maximum advantage of nanotechnology for biogas and methane production.
4. Microalgae and lignocellulose biomass are potential feedstock for bioenergy production. However, the effect of NPs on these substrates can be carried out for improvement in biogas production.
5. Other commonly applied methods for biogas escalation, including pretreatment of substrate or inoculum and supplementation biological and inorganic additives, can be used in combination nanomaterials to get an overall energy gain.

## Conclusion

By method of quantitative literature review, the impact of NMs on biogas production and methane yield is stated in this study. Several kinds of NMs have been investigated as additives in the AD process for biogas augmentation for various kinds of biodegradable wastes. For brevity, the eventual effect of nanomaterials and their positive or negative impacts on biogas generation are summarised in Table 8, which is concluded from the exhaustive literature review and presented from the materials’ point of view. Additionally, the following conclusions have been drawn from the reviewed literature.

• Metal NPs such as NZVI, Co., and Ni showed a positive effect on biogas yield. However, Ag NPs showed no inhibitory effect.
• Metal oxide NPs such as iron oxide (Fe2O3 and Fe3O4), Co3O4, NiO, MoO3 NPs showed an increase in biogas and methane production, whereas ZnO, TiO2, CeO2, and CuO NPs showed an inhibitory effect. In contrast, the literature showed MgO NPs showed a mixed effect.
• Nb-based compounds (NbO2, Nb3.49N4.56O0.44, and NbN) and nano-scale transition metal carbides (HfC, SiC, TiC, and WC) showed an enhancement in biogas yield.
• Carbon nanotubes showed a mixed effect. Single-walled CNTs showed no effect, whereas multiwall CNTs showed an increase in biogas production.

**TABLE 8:** Reported nanomaterials and their influence on biogas generation.

Category	Nanomaterials	Effect on biogas production
Metal Nanoparticles	NZVI, Co., Ni	Increase biogas production rate
	Ag, Au, Cu	Decrease or no change biogas production rate
Metal Oxide Nanoparticles	Fe_2_O_3_, Fe_3_O_4_, Co_3_O_4_, NiO, MoO_3_	Increase biogas production rate
	CeO_2_	Mixed-effect on biogas production depending upon size and concentration of NPs
	ZnO, CuO, TiO_2_, MgO, MnO_2_	Decrease or no change biogas production rate
Nano-scale Nb-based compounds	NbO_2_, Nb_3.49_N_4.56_O_0.44_, and NbN	Increase biogas production rate
Nano-scale transition metal carbides	HfC, SiC, TiC, WC	Increase biogas production rate
Carbon Nanotubes	SWCNTs	No change biogas production rate
	MWCNTs	Mixed-effect on biogas production depending upon size and concentration of NPs
Nanowires	Octahedral molecular sieve (OMS-2)	Increase biogas production rate
	ZnO Nanowire	No change biogas production rate
Nano-composite	Ni-Gr Nano -composite	Increase biogas production rate
Nano Ash	MNFA, MNBA	Increase biogas production rate

## Abbreviations

AD, Anaerobic Digestion; AGS, Anaerobic Granular Sludge; CM, Cattle Manure; COD, Chemical Oxygen Demand; DIET, Direct Interspecies Electron Transfer; EGSB, Expanded Granular Sludge Bed; EPS, Extracellular Polymeric Substance; HRT, Hydraulic Retention Time; ICZ, NZVI coated zeolite; MEG, Mono-Ethylene Glycol; MSW, Municipal Solid Waste; MWCNT, Multi-Walled Carbon Nanotubes; NC, Nano-composite; NM, Nanomaterials; NP, Nanoparticles; NW, Nanowires; NZVI, Nanoscaled Zero-Valent Iron; OMS, Octahedral Molecular Sieve; SWCNT, Single-Walled Carbon Nanotubes; TS, Total Solids; TSS, Total Suspended Solids; UASB, Up-flow Anaerobic Sludge Blanket; VFA, Volatile Fatty Acids; VS, Volatile Solids; WAS, Waste Activated Sludge.
